# Effectiveness and Adherence of Standalone Digital Tobacco Cessation Modalities: A Systematic Review of Systematic Reviews

**DOI:** 10.3390/healthcare13172125

**Published:** 2025-08-26

**Authors:** Maria Pia Di Palo, Federica Di Spirito, Marina Garofano, Rosaria Del Sorbo, Mario Caggiano, Francesco Giordano, Marianna Bartolomeo, Colomba Pessolano, Massimo Giordano, Massimo Amato, Alessia Bramanti

**Affiliations:** Department of Medicine, Surgery and Dentistry, University of Salerno, Via S. Allende, 84081 Baronissi, Italy; mgarofano@unisa.it (M.G.); rdelsorbo@unisa.it (R.D.S.); macaggiano@unisa.it (M.C.); frgiordano@unisa.it (F.G.); mbartolomeo@unisa.it (M.B.); cpessolano@unisa.it (C.P.); masgiordano@unisa.it (M.G.); mamato@unisa.it (M.A.); abramanti@unisa.it (A.B.)

**Keywords:** smoking, smoking cessation, smoke, tobacco, cancer prevention, public health, medication adherence, technology, telemedicine, digital tools

## Abstract

**Background:** The World Health Organization defined specific recommendations about digital tobacco cessation modalities as a self-management tool or as an adjunct to other support for adults. **Objectives**: The present umbrella review primarily aimed to assess the long-term (≥6 months) effectiveness and adherence of the different standalone digital tobacco cessation modalities (mobile text messaging, smartphone apps, Internet-based websites and programs, AI-based), administered individually or in combination; secondarily, the study aimed to assess the effect on smokers’ health. **Methods**: The present study (PROSPERO number: CRD42024601824) followed the PRISMA guidelines. The included studies were qualitatively synthesized and evaluated through the AMSTAR-2 tool. **Results**: Forty-five systematic reviews were included, encompassing 164,010 adult daily smokers of combustible tobacco. At 6 months, highly interactive or human-centered digital tools showed higher effectiveness (biochemically verified continuous abstinence rates (CARs) were 11.48% for smartphone apps and 11.76% for video/telephone counseling). In contrast, at 12 months, simpler, less interactive tools demonstrated higher effectiveness (self-reported CARs was 24.38% for mobile text messaging and 18.98% for Internet-based). Adherence rates were generally high, particularly with human-centered digital tools, amounting to 94.12% at 6 months and 64.08% at 12 months. Compared with individually administered digital tobacco cessation modalities, at 12 months, combined ones registered slightly higher effectiveness (self-reported CARs were 13.12% vs. 13.94%) and adherence (62.36% vs. 63.70%), potentially attributed to the multi-component nature and longer durations. **Conclusions**: Clinicians should prioritize combined digital tobacco cessation interventions that incorporate human-centered engagement initially, alongside simpler, sustained digital support to enhance long-term effectiveness and adherence. Future research should explore long-term medical and oral health benefits to assess the impact on overall health and well-being.

## 1. Introduction

The World Health Organization (WHO) has identified tobacco smoke as one of the biggest public health concerns, and it causes 7 million deaths from direct tobacco smoke and 1.3 million from secondhand tobacco smoke around the world every year [1].

As a consequence, smoking cessation interventions play a key role in public health, with short- and long-term positive effects on human health for subjects who quit smoking [2]. A six-month period of smoking abstinence was used as a temporal benchmark for long-term cessation, as this timeframe is considered a reliable indicator of long-term cessation, reflecting an estimated 50% likelihood of sustained abstinence in subsequent years [2].

In the era of technological advancement, which has driven a deeper transformation in healthcare services [3], several new digital tobacco cessation modalities have also been widespread in low- and middle-income countries [4], including interventions based on low-tech methods, such as mobile text messaging, to more complex and recently high-tech methods, such as those based on artificial intelligence (AI) [5,6].

In fact, specific recommendations have been defined by the WHO in the 2024 clinical guidelines for tobacco cessation in adults [7] about the use of digital tobacco cessation modalities as behavioral support. According to the WHO, digital interventions for tobacco cessation, individually or in combination, can be provided to adult smokers who want to quit, either as a self-management tool or as an adjunct to other tobacco cessation support. These recommendations on digital tobacco cessation modalities were defined as conditional, which means that the balance between desirable effects outweighs the undesirable ones, and this difference justified that following the recommendation is favored, although the degree of this preference had a modest margin [7].

An umbrella review published in 2025 [8] evaluated the long-term effectiveness (≥6 months) and adherence of digital tobacco cessation modalities provided as an adjunct to both pharmacological and non-pharmacological alternative tobacco cessation support. The study showed that digital tobacco cessation support for other non-pharmacological modalities had similar effectiveness but higher adherence compared with digital tobacco cessation support for pharmacological modalities at 6 months (the continuous abstinence rates were 14.85% vs. 9.06% and adherence rates were 83.43% vs. 41.37%, respectively); instead, the effectiveness was similar, but adherence was lower at 12 months (the continuous abstinence rates were 9.08% vs. 8.51% and adherence rates were 66.59% vs. 83.92%, respectively) [8].

In contrast, the evidence on the long-term effectiveness (≥6 months) and adherence of standalone digital tobacco cessation modalities was lacking and based on limited certainty [7]. The WHO identified four categories of digital tobacco cessation modalities: mobile text messaging, smartphone applications (apps), Internet-based websites and programs, and AI-based interventions [7].

Mobile text messaging as a standalone digital tobacco cessation had the strongest evidence, and it was the only one for which the recommendation was based on moderate certainty, probably due to its older digital nature [7]. Smartphone apps were widely considered in tailoring, engagement, and interactivity [9], but the recommendation was low [7]. Internet-based websites and programs had a lower level of certainty (very low), a result of their more challenging use [7]. Finally, AI-based interventions are rapidly evolving, but at present still have a low level of certainty [7].

Another shortcoming in the currently available evidence concerns the combination of different digital tobacco cessation modalities. In fact, although the WHO guidelines mention individual or combined digital tobacco cessation modalities, there is no evidence on the different combinations and the related long-term effectiveness (≥6 months) and adherence.

The assessment of the impact of standalone digital tobacco cessation modalities requires taking into consideration the intervention adherence, which plays a key role in influencing the overall effectiveness. In fact, effectiveness and adherence have a bidirectional relationship known as “reverse causality” [10,11], whereby not only does adherence influence the effectiveness, but the perceived success or lack thereof also affects the smoker’s motivation to continue the smoking cessation program. For instance, low adherence can decrease the intervention’s effectiveness, but also early releases or the feeling of failure might further decrease the intervention, leading to reduced adherence in treatment over time. Not considering this bidirectional relationship could cause an overestimation of the adherence influence on intervention effectiveness [10,11].

Therefore, while the previous umbrella review (2025) [8] was focused exclusively on digital tobacco cessation modalities as an adjunct to other tobacco cessation support, the present study explores standalone modalities used as a self-management method, as this has also been recommended by the WHO guidelines [7]. Given the variety of existing systematic reviews with differing scopes, quality, and digital tobacco cessation interventions categorizations [8], an umbrella review approach was adopted to collate and critically appraise this highest-level evidence, providing a consolidated view for policy and practice and reorganizing the findings of the previous systematic review according to the WHO 2024 classification of digital tobacco cessation interventions [7].

In particular, the present systematic review of systematic reviews primarily aimed to assess the long-term effectiveness (≥6 months) and adherence of the different standalone digital tobacco cessation modalities (mobile text messaging, smartphone apps, Internet-based websites and programs, and AI-based interventions), when administered individually or in combination.

Furthermore, in consideration of the public health issue of tobacco smoke, which causes tobacco-related noncommunicable diseases (cardiovascular [12], pneumological [13], metabolic [14], and oral diseases [15]) and impairment of quality of life and mental health [16], often in a dose-dependent manner, the present study secondarily aimed to assess the effect of digital tobacco cessation modalities on smokers’ health.

## 2. Materials and Methods

### 2.1. Study Protocol

Before conducting the literature searches, the study protocol was registered (registration number: CRD42024601824) in PROSPERO (International Prospective Register of Systematic Reviews) [17].

The research questions were: “What is the long-term effectiveness (≥6 months) and adherence of the different standalone digital tobacco cessation modalities (mobile text messaging, smartphone apps, Internet-based websites and programs, AI-based interventions), individually or combined, on adult (≥18 years old) smokers? What are the related long-term smoking cessation effects on smokers’ health?”

The following PICO model [18] was established to develop the research questions, search strategy, and the inclusion and exclusion criteria:

- Population (P): Current daily adult (≥18 years old) smokers of combustible tobacco, as referred to by the WHO [19];
- Intervention (I): Standalone individually administered digital tobacco cessation modalities (mobile text messaging, smartphone apps, Internet-based websites and programs, AI-based interventions) from all providers and setting types;
- Comparison (C): Standalone combined administered digital tobacco cessation modalities (combination of mobile text messaging and/or smartphone apps and/or Internet-based websites and programs and/or AI-based interventions) from all providers and setting types;
- Outcomes (O):
  - Primary outcome(s): Point prevalence abstinence (PPA) and/or continuous abstinence rates (CARs) at least ≥6 months from the start of the digital tobacco cessation intervention, biochemically verified (e.g., cotinine or carbon monoxide test) and/or self-reported, as referred to the Russell Standard and the Society for Research on Nicotine and Tobacco [20,21].
  If reported, the secondary outcome(s) extracted were:
  - Secondary outcome(s): Adherence, satisfaction, and acceptability to digital tobacco cessation modalities; medical (cardiovascular/pneumological/metabolic/psychological) and oral (periodontal/peri-implant/mucosal lesions) parameters before and after digital tobacco cessation modalities.

### 2.2. Search Strategy

Both the electronic and manual searches were carried out without any filter to restrict the date of studies’ publication to until 10 October 2024 to retrieve all relevant English systematic reviews on standalone digital tobacco cessation modalities (any) reporting primary outcomes evaluating smoking cessation rates in at least 6 months after the beginning of the intervention.

Three electronic databases (MEDLINE/PubMed, Scopus, and Web of Science Core Collection) and the PROSPERO register were consulted by three reviewers (M.P.D.P., F.D.S., and A.B.), who worked in duplicate and independently using the following search strategy combined with Boolean operators and available filters (Table 1):

### 2.3. Study Selection and Eligibility Criteria

After the establishment of the eligibility criteria, the records retrieved from the electronic searches in the databases were collected and screened by two reviewers (M.P.D.P. and F.D.S.), who worked in duplicate and independently. The issue of disagreement in any step of the study selection process was resolved by discussing with a third reviewer (A.B.). The first step of the study selection process consisted of the removal of duplicate records. The second step consisted of the screening of the remaining title–abstracts to eliminate the records not eligible based on the aforementioned purpose. The third step consisted of screening the remaining full texts to remove the records that did not comply with the inclusion criteria described below.

The fourth step consisted of the additional manual search, which was performed by screening the bibliography of the systematic reviews included from the electronic searches.

The 2.80.1 version of the Mendeley Reference Manager tool was used to collect all references of the included records.

Inclusion criteria were: systematic reviews with and without meta-analysis published in English without restrictions concerning date of publications, which evaluated the long-term effectiveness (≥6 months after the start of the smoking cessation interventions) of the different standalone digital tobacco cessation modalities (mobile text messaging, smartphone apps, Internet-based websites and programs, AI-based interventions), administered individually or in combination, to adult (≥18 years old) current daily combustible tobacco products smokers who did not drink alcohol or have disorders of substance abuse.

No restrictions were applied based on the design or number of studies included in the included systematic reviews, the sample size or the comorbidities of the population, the characteristics of the smoking behaviors (number of cigarettes smoked per day or the combustible tobacco smoked types), and the type of digital tobacco cessation modalities.

Exclusion criteria were: not English systematic reviews; previously updated systematic reviews (only the most recent updated systematic review was included); under 18 years old smokers, pregnant woman, lactating woman, smokers drinking alcohol or with disorders of substance abuse; not current daily smokers nor smokers of combustible tobacco products; not standalone digital tobacco cessation modalities (e.g., digital tobacco cessation modalities combined with pharmacological or not digital-based behavioral interventions); and standalone digital tobacco cessation modalities used only to assess primary outcomes at the follow-ups or for less than 6 months after the beginning of the intervention.

### 2.4. Data Extraction and Collection

The data of the included systematic reviews were collected and extracted by two reviewers (M.P.D.P. and F.D.S.) who worked in duplicate and independently in a standardized data extraction form based on the proposed model for intervention reviews of non-randomized clinical trials and randomized clinical trials [22]. The issue of disagreement in any step of the data extraction and collection process was resolved by discussing with a third reviewer (A.B.).

When systematic reviews reported data for more than one standalone digital tobacco cessation modality, the number of participants and the related variables and outcome measures were extracted separately for each intervention category (e.g., mobile text messaging, smartphone apps, AI-based interventions). This procedure ensured that participants from the same systematic review were allocated only once within the relevant category, avoiding duplication of sample sizes or outcomes. The extracted data from all reviews were then collected, synthesized, and analyzed within each category to allow a descriptive comparison across intervention types.

The data from the included systematic reviews, which were collected and extracted, were:

- Study features: First author, year, journal, included study’s number and design, meta-analysis or no meta-analysis, assessed quality, funding information (if any);
- Population features: Sample size (n.), mean and/or range age, gender ratio (male/female), comorbidities, smoked cigarettes per day, nicotine addiction severity, quit smoking motivation;
- Intervention and comparison features: Type and duration of digital tobacco cessation modalities;
- Outcome(s):
  - Primary outcome(s): PPA and/or CARs, smoked cigarettes per day, failure reasons (if any);
  - Secondary outcome(s): Adherence, satisfaction, and acceptability to digital tobacco cessation modalities; medical (cardiovascular/pneumological/metabolic/psychological) and oral (periodontal/peri-implant/mucosal lesions) parameters before and after digital tobacco cessation modalities.

For studies reporting both complete case and intention-to-treat (ITT) results for smoking cessation, only ITT rates were used to ensure that all enrolled participants were included in the analysis, irrespective of the intervention completion or follow-up. In line with ITT methodology, participants who dropped out or who were lost to follow-up were assessed as smokers. The ITT analysis was recommended for evaluating the effectiveness of an intervention [23].

Only the data about standalone digital tobacco cessation modalities followed up for at least ≥6 months for adult (≥18 years old) current daily combustible tobacco product smokers were collected and extracted.

### 2.5. Data Synthesis

The collected and extracted data were qualitatively synthesized in a worksheet of Microsoft Excel software 2019 (Microsoft Corporation, Redmond, WA, USA) by means of descriptive statistical analysis to:

- Evaluate long-term effectiveness (≥6 months) of the different standalone digital tobacco cessation modalities (mobile text messaging, smartphone apps, Internet-based websites and programs, AI-based interventions);
- Compare long-term effectiveness (≥6 months) of individual vs. combined standalone digital tobacco cessation modalities;
- Evaluate adherence/satisfaction/acceptability of the different standalone digital tobacco cessation modalities (mobile text messaging, smartphone apps, Internet-based websites and programs, AI-based interventions);
- Compare adherence/satisfaction/acceptability of the different standalone digital tobacco cessation modalities;
- Evaluate long-term effectiveness (≥6 months) of digital tobacco cessation modalities on medical (cardiovascular/pneumological/metabolic/psychological) and oral (periodontal/peri-implant/mucosal lesions) parameters before and after digital tobacco cessation modalities;
- Compare long-term effectiveness (≥6 months) of digital tobacco cessation modalities on medical (cardiovascular/pneumological/metabolic/psychological) and oral (periodontal/peri-implant/mucosal lesions) parameters before and after individual vs. combined different digital tobacco cessation modalities.

### 2.6. Quality Assessment and Overlap Management

The qualitative assessment of the included systematic reviews was judged by two reviewers (M.P.D.P. and F.D.S.), who worked in duplicate and independently on 18 November 2024, with the Assessing the Methodological quality of Systematic Reviews-2 (AMSTAR-2) tool [24]. The issue of disagreement in any step of the quality assessment process was resolved through discussion with a third reviewer (A.B.).

To assess the degree of primary studies overlap between the included systematic reviews, the corrected cover area (CCA) was calculated as recommended by Pieper et al. [25]. A degree between 0 and 5% was judged as “slight”, between 6 and 10% as “moderate”, between 11 and 15% as “high”, or >15% as “very high” [25].

## 3. Results

### 3.1. Study Selection

A total of 931 records were collected by the electronic searches of the PROSPERO register (*n* = 322), PubMed/MEDLINE (*n* = 195), Scopus (*n* = 268), and Web of Science Core Collection (*n* = 146). The first step of the study selection process allowed the removal of 234 duplicate records. The second step consisted of screening the remaining 697 title–abstracts, which allowed the elimination of 411 records not eligible based on the aforementioned purpose.

The third step consisted of screening the remaining 286 full texts, which allowed the removal of 248 records that did not comply with the inclusion criteria for the following exclusion reasons: not smoking cessation data (or impossibility to extract) at follow-up of at least ≥6 months (*n* = 69); ongoing systematic reviews (*n* = 65); not standalone digital tobacco cessation modalities (or impossibility to extract data) (*n* = 63); age population < 18 years, or range age not defined, or impossibility to extract data of smokers ≥18 years (*n* = 30); previous updated systematic reviews or not systematic reviews (*n* = 18); systematic reviews not in English (*n* = 1); pregnant woman (*n* = 1); not current daily smokers of combustible tobacco (*n* = 1).

The corresponding author of one record with the full text not available was contacted via email to obtain and screen the full text. However, as no response was received, the record (*n* = 1) was excluded.

A total of 37 records from the electronic searches were included [26,27,28,29,30,31,32,33,34,35,36,37,38,39,40,41,42,43,44,45,46,47,48,49,50,51,52,53,54,55,56,57,58,59,60,61,62].

The fourth step consisted of the manual search, which was performed using the same methodology as the study selection process of the electronic search and by screening the bibliography of the systematic reviews included. A total of 2089 records from the manual search were screened, and 359 duplicate records were removed. During the screening of the remaining 1730 title–abstracts, 1665 records were eliminated because they were not compliant with the aforementioned purpose.

During the reading of the remaining 65 full texts, which were available for all records, 57 records were excluded based on the eligibility criteria for the following reasons: not smoking cessation data (or impossibility to extract) at follow-up of at least ≥6 months (*n* = 18); not standalone digital tobacco cessation modalities (or impossibility to extract data) (*n* = 13); age population < 18 years, or range age not defined, or impossibility to extract data of smokers ≥18 years (*n* = 12); previous updated systematic reviews or not systematic reviews (*n* = 14).

A total of eight records from the manual searches were included [63,64,65,66,67,68,69,70].

**Figure 1:**
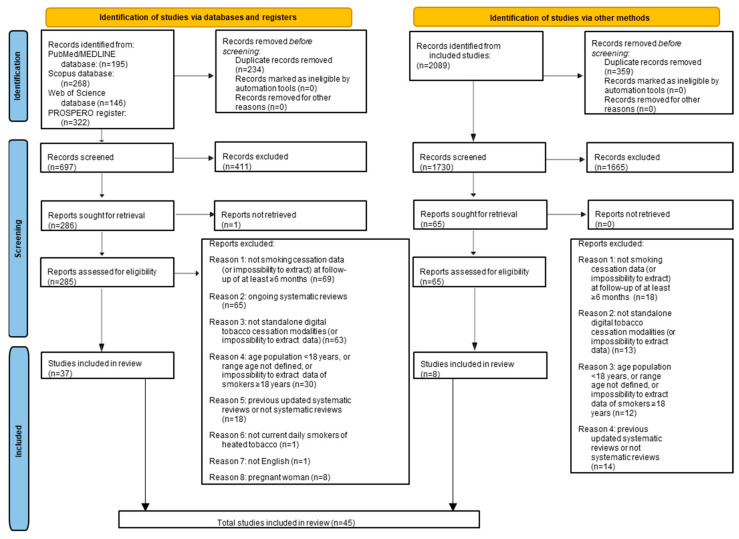
PRISMA flow chart 2020 showing the number of records identified, screened, excluded, and finally included at each stage of the electronic and manual study selection process.

Therefore, 45 systematic reviews [26,27,28,29,30,31,32,33,34,35,36,37,38,39,40,41,42,43,44,45,46,47,48,49,50,51,52,53,54,55,56,57,58,59,60,61,62,63,64,65,66,67,68,69,70] were included (Figure 1).

### 3.2. Study Characteristics and Qualitative Synthesis

Data from 45 systematic reviews [26,27,28,29,30,31,32,33,34,35,36,37,38,39,40,41,42,43,44,45,46,47,48,49,50,51,52,53,54,55,56,57,58,59,60,61,62,63,64,65,66,67,68,69,70] are extracted in Table S1 of the Supplementary File S1, which illustrates study characteristics; Table S2 of the Supplementary File S2 illustrates the digital tobacco cessation modalities’ features.

A total of 178 studies (168 randomized controlled trials, 4 cohort studies, 3 prospective studies, 2 pilot studies, and 1 observational study) were included in the 45 systematic reviews [26,27,28,29,30,31,32,33,34,35,36,37,38,39,40,41,42,43,44,45,46,47,48,49,50,51,52,53,54,55,56,57,58,59,60,61,62,63,64,65,66,67,68,69,70], 24 with a meta-analysis [27,28,29,33,35,36,40,41,43,47,53,54,57,59,60,61,62,63,65,66,67,68,69,70] and 21 without a meta-analysis [26,29,31,32,34,37,38,39,42,44,45,46,48,49,50,51,52,55,56,58,64].

### 3.3. Individual Digital Tobacco Cessation Modalities

The smoking cessation strategies based on individual digital modalities were performed on 142,069 smokers; of these, 1936 (1.36%) smokers were hospitalized for undefined diseases, 544 (0.38%) were affected by psychiatric disorders, 312 (0.22%) were outpatients for pre-surgery or diagnostic procedures, 80 (0.06%) were affected by tuberculosis, and 46 (0.03%) were hospitalized for acute myocardial infarction. The mean age was reported for 35,733 smokers and was 38.70 years old, while the gender ratio was 17,884 males to 22,953 females (1:1.28).

The average number of cigarettes smoked per day before the smoking cessation intervention was reported for 9341 smokers and was 13.51. The recorded FTND was low in 112 subjects, moderate in 63, and high in 59.

The 6-month CARs biochemically verified at 6 months were recorded for 5713 smokers and amounted to 417 (7.30%) former smokers; while the self-reported amount was recorded for 19,228 smokers and amounted to 1648 (8.57%) former smokers.

The 12-month CARs self-reported at 12 months were recorded for 8523 smokers and amounted to 1118 (13.12%) former smokers.

The 7-day PPA biochemically verified at 6 months was recorded for 5663 smokers and amounted to 458 (8.09%) former smokers; while the self-reported amount was recorded for 34,644 smokers and amounted to 7396 (21.35%) former smokers.

The 7-day PPA biochemically verified at 12 months was recorded for 1577 smokers and amounted to 84 (5.33%) former smokers, while the self-reported amount was recorded for 20,206 smokers and amounted to 3789 (18.75%) former smokers.

The adherence rate was 73.72% at 6 months (the smoking status of 38,728 subjects was assessed) and 62.36% at 12 months (the smoking status of 12,990 subjects was assessed).

**Figure 2:**
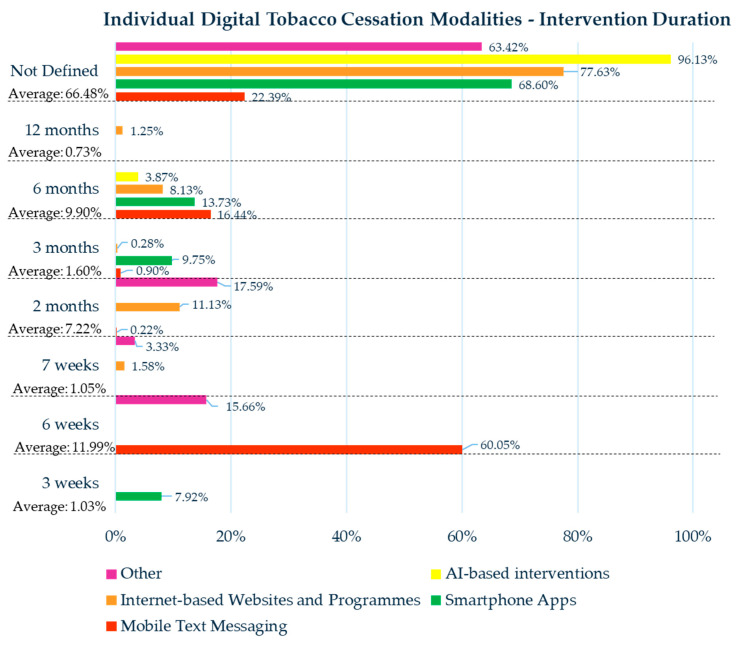
Duration lengths of standalone individual digital tobacco cessation modalities.

The duration lengths of individual digital tobacco cessation modalities are displayed in Figure 2.

#### 3.3.1. Mobile Text Messaging

The smoking cessation strategy based on mobile text messaging modalities was performed on 27,028 smokers [28,34,36,39,40,51,57,59,60,62,65], where 317 of them were hospitalized patients for an undefined disease [34].

The 6-month CARs verified by cotinine test at 6 months were recorded in 2738 smokers and amounted to 138 (5.04%) former smokers [39,59], while self-reported amounts were recorded in 2498 smokers and amounted to 237 (9.49%) former smokers [28,40,59,62].

The 12-month CARs self-reported at 12 months were recorded in 320 smokers and amounted to 78 (24.38%) former smokers [40,59].

The 7-day PPA verified by carbon monoxide tests at 6 months was recorded in 1509 smokers and amounted to 96 (6.36%) former smokers [40], while the self-reported amount was recorded in 20,226 smokers and amounted to 5135 (25.39%) former smokers [28,36,39,40,59,60].

The adherence rate was 83.76% at 6 months (the smoking status of 9312 subjects was assessed) [39,40,59,60].

#### 3.3.2. Smartphone Apps

The smoking cessation strategy based on smartphone app was performed on 18,448 smokers [32,33,35,36,40,41,42,44,48,51,55,59,60,62]; the mean age was reported for 5312 smokers and was 36.28 years old [26,32,33,40,41,42,48,51,55,59,60,62,63], while the reported gender ratio was 4969 males to 5263 females (1:1.06) [26,32,33,40,41,42,44,48,51,55,59,60,62,63].

The smoking status before the intervention was recorded for 3260 subjects, who smoked a mean of 17.2 cigarettes per day [26,32,40,41,42,60,61,63].

The 6-month CARs verified by carbon monoxide tests at 6 months were recorded in 1960 smokers and amounted to 225 (11.48%) former smokers [40,41,42,44,59,60], while the self-reported amounts were recorded in 10,070 smokers and amounted to 890 (8.84%) former smokers [26,32,33,35,36,40,41,42,48,51,55,59,60,62,63].

The 7-day PPA verified by a carbon monoxide test at 6 months was recorded in 1600 smokers and amounted to 144 (9.00%) former smokers [14,26,59,63], while the self-reported amount was recorded in 7452 smokers and amounted to 1308 (17.55%) former smokers [26,33,36,48,51,55,59,62].

The 7-day PPA self-reported at 12 months was recorded in 4830 smokers and amounted to 1316 (27.24%) former smokers [32,36].

The adherence rate was 43.17% at 6 months (the smoking status of 5809 subjects was assessed) [26,32,33,35,36,40,41,42,48,51,55,59,60,62,63].

#### 3.3.3. Internet-Based Websites and Programs

The smoking cessation strategies based on Internet-based websites and programs digital modalities were performed on 83,399 smokers [28,32,35,36,38,40,41,43,47,48,49,52,53,54,57,58,59,60,61,64,65,66,67], 544 (0.65%) of whom were affected by psychiatric disorders [40,52,54]. The mean age was reported for 29,201 smokers and was 38.55 years old, while the gender ratio was 12,702 males to 17,497 females (1:1.38) [28,38,40,43,47,49,52,57,58,61,64,65,66,67].

The average number of cigarettes smoked per day before the smoking cessation intervention was reported for 4780 smokers and was 11.62. The recorded FTND was low in 112 subjects, moderate in 63, and high in 59 [40,43,47,48,52,57,58,64,65,66,67].

The 6-month CARs biochemically verified at 6 months were recorded for 964 smokers and amounted to 48 (4.98%) former smokers [35,41,59,60], while the self-reported amount was recorded for 3534 smokers and amounted to 388 (10.98%) former smokers [43,57,58,65].

The 12-month CARs self-reported at 12 months were recorded for 3703 smokers and amounted to 703 (18.98%) former smokers [40,43,57,65,67].

The 7-day PPA biochemically verified at 6 months was recorded for 2554 smokers and amounted to 218 (8.54%) former smokers [35,40,41,43,47,48,52,54,57,58,59,60,65], while the self-reported amount was recorded for 4473 smokers and amounted to 697 (15.58%) former smokers [36,43,48,58].

The 7-day PPA biochemically verified at 12 months was recorded for 1577 smokers and amounted to 84 (5.33%) former smokers [38,48,49,58,61,67], while the self-reported amount was recorded for 14,160 smokers and amounted to 2103 (14.58%) former smokers [32,43,48,57,58,65].

The adherence rate was 70.09% at 6 months (the smoking status of 27,101 subjects was assessed) [28,35,40,41,43,47,52,54,57,58,59,60,65] and 62.39% at 12 months (the smoking status of 9167 subjects was assessed) [40,43,57,65,66].

#### 3.3.4. AI-Based Interventions

The smoking cessation strategies based on individual digital modalities were performed on 8055 smokers [27,32,34,43,50,51,57,58,65,68], 814 (10.11%) of whom were hospitalized for not undefined diseases [34], and 312 (3.87%) smokers were outpatients for pre-surgery or diagnostic procedures [50,68]. The mean age was reported for 406 smokers and was 32.8 years old [27], while the gender ratio was 213 males to 193 females (1.10:1) [27,58].

The average number of cigarettes smoked per day before the smoking cessation intervention was reported for 1301 smokers and was 11.21 [57,58].

The 6-month CARs self-reported at 6 months were recorded for 1982 smokers and amounted to 55 (2.77%) [43].

The 12-month CARs self-reported at 12 months were recorded for 1548 smokers and amounted to 139 (8.98%) former smokers [43,57,65].

The 7-day PPA self-reported at 6 months was recorded for 2447 smokers and amounted to 236 (9.64%) former smokers [51,57,58].

The 7-day PPA self-reported at 12 months was recorded for 584 smokers and amounted to 181 (30.99%) former smokers [32].

The adherence rate was 42.58% at 6 months (the smoking status of 458 subjects was assessed) [57] and 59.72% at 12 months (the smoking status of 1579 subjects was assessed) [43,57,65].

#### 3.3.5. Other Digital Tobacco Modalities

The smoking cessation strategies based on other digital modalities were performed on 5139 smokers [29,31,32,34,36,40,43,57,62,65,70], 805 (15.66%) of whom were hospitalized for not undefined diseases [34]; a total of 80 (1.56%) smokers were affected by tuberculosis [36], and 46 (0.90%) were hospitalized for acute myocardial infarction [34]. The mean age was reported for 5139 smokers and was 55.87 years old [57,62,68].

The 6-month CARs biochemically verified at 6 months were recorded for 51 smokers and amounted to 6 (11.76%) former smokers [70]; while the self-reported amount was recorded for 904 smokers and amounted to 78 (8.63%) former smokers [57,68].

The 12-month CARs self-reported at 12 months were recorded for 2953 smokers and amounted to 198 (6.71%) former smokers [43,57,65,68].

The 7-day PPA self-reported at 6 months was recorded for 46 smokers and amounted to 20 (43.48%) former smokers [34].

The 7-day PPA self-reported at 12 months was recorded for 632 smokers and amounted to 189 (29.91%) former smokers [32,34].

The adherence rate was 94.12% at 6 months (the smoking status of 51 subjects was assessed) [57,65,70] and 64.08% at 12 months (the smoking status of 2244 subjects was assessed) [57,65,70].

Table 2 shows all the outcomes of effectiveness, adherence, and satisfaction reported for the individual digital tobacco cessation modalities.

### 3.4. Combined Digital Tobacco Cessation Modalities

The smoking cessation strategies based on combined digital modalities were performed on 21,941 smokers; a total of 1004 (4.58%) smokers were affected by schizoaffective disorders, bipolar disorders, 541 were outpatients for pre-surgery or diagnostic procedures (2.47%), and 726 (3.31%) were in inpatient rehabilitation centers. The mean age was reported for 8656 smokers and was 38.11 years old, while the gender ratio was 2904 males to 5752 females (1:1.98).

The average number of cigarettes smoked per day before the smoking cessation intervention was reported for 4199 smokers and was 12.22; the number after the intervention was registered in 180 and was 10.46.

The 6-month CARs biochemically verified at 6 months were recorded for 1048 smokers and amounted to 116 (11.07%) former smokers, while the self-reported amount was recorded for 3631 smokers and amounted to 566 (15.59%) former smokers.

The 12-month CARs self-reported at 12 months were recorded for 5380 smokers and amounted to 750 (13.94%) former smokers.

The 7-day PPA biochemically verified at 6 months was recorded for 405 smokers and amounted to 130 (32.10%) former smokers, while the self-reported amount was recorded for 3388 smokers and amounted to 716 (21.13%) former smokers.

The 7-day PPA self-reported at 12 months was recorded for 7426 smokers and amounted to 1018 (13.71%) former smokers.

The adherence rate was 67.59% at 6 months (the smoking status of 4218 subjects was assessed) and 63.70% at 12 months (the smoking status of 2325 subjects was assessed).

**Figure 3:**
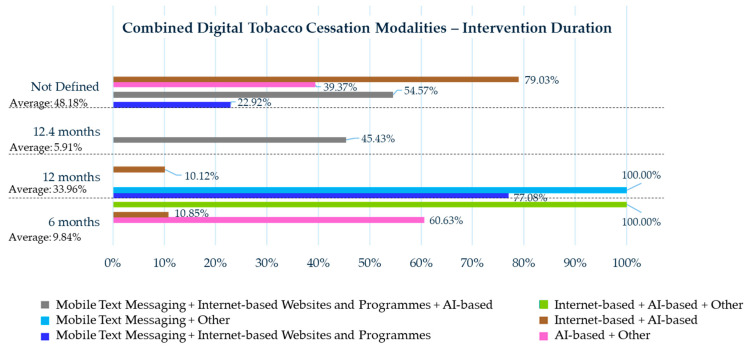
Duration lengths of standalone combined digital tobacco cessation modalities.

The duration lengths of combined digital tobacco cessation modalities are displayed in Figure 3.

#### 3.4.1. Mobile Text Messaging Plus Internet-Based Websites and Programs

The smoking cessation strategy based on Internet-based plus mobile text messaging was performed on 3334 smokers [36,43,52], 783 of which were affected by schizoaffective disorders [52], and 221 by bipolar disorders [52]; the mean age was reported for 3023 smokers and was 47.07 years old, while the gender ratio was 701 males to 2322 females (1:3.31) [36,43,52].

The 12 months CARs self-reported at 12 months were recorded for 453 smokers and amounted to 24 (5.30%) former smokers [43].

#### 3.4.2. Mobile Text Messaging Plus Other

The smoking cessation strategy based on telephone counseling plus mobile text messaging was performed on 3852 smokers [46,51].

The 6-month CARs self-reported at 6 months were recorded for 3631 smokers and amounted to 566 (15.59%) former smokers [51].

The 12-month CARs self-reported at 12 months were recorded for 3631 smokers and amounted to 465 (12.81%) former smokers [51].

#### 3.4.3. Mobile Text Messaging Plus Internet-Based Websites and Programs Plus AI-Based Interventions

The smoking cessation strategy based on Internet-based websites and programs plus mobile text messaging plus AI-based intervention was performed on 2853 smokers [35,37,40,41,43,53,57,59,60,65,66,67]; the mean age was reported for 1296 smokers and was 39.5 years old, while the gender ratio was 648 males to 648 females (1:1), smoking a mean of 16.6 cigarettes per day [37,40,41,43,53,57,65,66,67].

The 6-month CARs verified by a cotinine test at 6 months were recorded for 1048 smokers and amounted to 116 (11.07%) [35,41,59,60].

The 12-month CARs self-reported at 12 months were recorded for 1296 smokers and amounted to 261 (20.14%) former smokers [37,40,41,43,53,57,65,66,67].

The 7-day PPA self-reported at 6 months was recorded for 1296 smokers and amounted to 378 (29.17%) former smokers [37,40,41,43,53,57,65,66,67], while the amount at 12 months amounted to 423 (32.64%) former smokers [37,40,41,43,53,57,65,66,67].

The adherence rate was 72.14% at 6 months (the smoking status of 756 subjects was assessed) [35,41,59,60] and 81.94% at 12 months (the smoking status of 1062 subjects was assessed) [37,40,41,43,53,57,65,66,67].

#### 3.4.4. Smartphone App Plus Other

The smoking cessation strategy based on a smartphone app plus computer-based video counseling plus mobile carbon monoxide checker was performed on 58 smokers [30].

The adherence rate was 98.28% (the smoking status of 57 was assessed) at 6 months [30].

#### 3.4.5. AI-Based Plus Other

The smoking cessation strategy based on telephone counseling plus AI-based intervention was performed on 541 smokers [50,68,69], 328 of which were outpatients for pre-surgery or diagnostic procedures [50,68].

The smoking cessation status before the intervention was recorded for 328 subjects who smoked at least twenty cigarettes per day, and 82 (25.00%) of them reduced the number of cigarettes smoked per day at 12 months by more than 50% (15.8 cigarettes per day as verified by a carbon monoxide test) [50,68].

#### 3.4.6. Internet-Based Plus AI-Based

The smoking cessation strategy based on Internet-based plus AI-based intervention was performed on 10,172 smokers [32,43,47,53,56,57,58,64,65,66,67]; the mean age were reported for 3932 smokers and was 32.55 years old, while the gender ratio was 1335 males to 2597 females (1:1.95) [47,53,57,58,64,65,67].

The smoking cessation status before the intervention was recorded for 2903 subjects, who smoked a mean of 10.27 cigarettes per day [47,53,57,58,64,65,67]. After the intervention, 98 smokers reduced the mean number of cigarettes per day to six cigarettes [32,56].

The 7-day PPA self-reported at 6 months was recorded for 1366 smokers and amounted to 167 (12.23%) former smokers [32,34,56,65,67], while the amount at 12 months was recorded for 6130 smokers and amounted to 595 (9.71%) former smokers [43,57,65,66].

The adherence rate was 52.09% (the smoking status of 1032 subjects was assessed) at 6 months and 40.72% at 12 months (the smoking status of 419 subjects was assessed) [4].

#### 3.4.7. Internet-Based Plus Other

The smoking cessation strategy based on website resources plus the creation of a personal video message was performed on 405 smokers with a mean age of 20.42 years, and the gender ratio was 220 males to 185 females (1.19:1) [40,47,57,58,65].

The 7-day PPA verified by acarbon monoxide test at 6 months amounted to 130 (32.10%) former smokers [40,47,57,58,65].

The adherence rate was 90.6% at 6 months (the smoking status of 367 subjects was assessed) [40,47,57,58,65].

#### 3.4.8. Internet-Based Plus AI-Based Plus Other

Internet-based plus AI-based plus computer-based counseling was performed on 726 smokers, all of whom were in inpatient rehabilitation centers [43,57,65].

The 7-day PPA self-reported at 6 months amounted to 171 (23.55%) former smokers [43,57,65].

The adherence rate was 88.43% at 6 months (the smoking status of 639 subjects was assessed) [43,57,65].

Table 3 shows all the outcomes of effectiveness, adherence, and satisfaction reported for the combined digital tobacco cessation modalities.

### 3.5. Overall: Individual and Combined Standalone Digital Tobacco Cessation Modalities

The standalone digital tobacco cessation modalities were performed on 164,010 smokers; a total of 1936 (1.18%) smokers were hospitalized for not undefined diseases, 1548 (0.94%) were affected by psychiatric disorders, 853 (0.52%) were outpatients for pre-surgery or diagnostic procedures, 726 (0.44%) were in inpatient rehabilitation centers, 80 (0.05%) were affected by tuberculosis, and 46 (0.03%) were hospitalized for acute myocardial infarction. The mean age was reported for 44,389 smokers and was 38.58 years old, while the gender ratio was 20,788 males to 28,705 females (1:1.38).

The average number of cigarettes smoked per day before the smoking cessation intervention was reported for 13,540 smokers and was 13.11, while the amount after the intervention was registered in 180 and was 10.46. The recorded FTND was low in 112 subjects, moderate in 63, and high in 59.

The 6-month CARs biochemically verified at 6 months were recorded for 6761 smokers and amounted to 533 (7.88%) former smokers, while the self-reported amount was recorded for 22,859 smokers and amounted to 2214 (9.69%) former smokers.

The 12-month CARs self-reported at 12 months were recorded for 13,903 smokers and amounted to 1868 (13.44%) former smokers.

The 7-day PPA biochemically verified at 6 months was recorded for 6068 smokers and amounted to 588 (9.69%) former smokers; while the self-reported amount was recorded for 38,032 smokers and amounted to 8112 (21.33%) former smokers.

The 7-day PPA biochemically verified at 12 months was recorded for 1577 smokers and amounted to 84 (5.33%) former smokers; while the self-reported amount was recorded for 27,632 smokers and amounted to 4807 (17.40%) former smokers.

The adherence rate was reported for 37,210 subjects and was 65.97% at 6 months (the smoking status of 56,402 subjects was assessed at 6 months follow-up), and it was also reported for 9581 subjects and was 62.56% at 12 months (the smoking status of 15,315 subjects was assessed at 12 months follow-up).

Table 4 shows all the outcomes of effectiveness, adherence, and satisfaction reported for the individual vs. combined digital tobacco cessation modalities, as well as the overall outcomes of the present study (individual plus combined).

Supplementary File S3 shows the findings recorded for each subgroup of the different digital tobacco cessation modalities. Table S3 clusters the data extracted for each individual digital tobacco cessation modality (intervention), Table S4 clusters for each combined digital tobacco cessation modality (comparison), and Table S5 summarizes the overall individual vs. combined digital tobacco cessation modality data, as well as the overall data extracted.

No data were available about quit smoking motivation or failure reason in quitting, acceptability, medical (cardiovascular/pneumological/metabolic/psychological), and oral (periodontal/peri-implant/mucosal lesions) parameters before and after digital tobacco cessation modalities.

### 3.6. Quality Assessment and Overlap Management

A total of 45 systematic reviews were judged using the AMSTAR-2 tool as follows: 13 (28.89% of the included systematic reviews) [27,29,30,43,45,47,54,57,59,60,68,69,70] were “high quality”, 4 (8.89%) [32,34,36,56] were “moderate quality”, 11 (24.44%) [26,28,33,38,39,40,44,58,63,65,67] were “low quality”, and 17 (37.78%) [31,35,37,41,42,46,48,49,50,51,52,53,55,61,62,64,66] were “critically low quality”.

The results for each of the 16 items of the risk of bias assessment and the related quality judgment of each of the 45 included systematic reviews [26,27,28,29,30,31,32,33,34,35,36,37,38,39,40,41,42,43,44,45,46,47,48,49,50,51,52,53,54,55,56,57,58,59,60,61,62,63,64,65,66,67,68,69,70] are reported in the Supplementary File S4.

**Figure 4:**
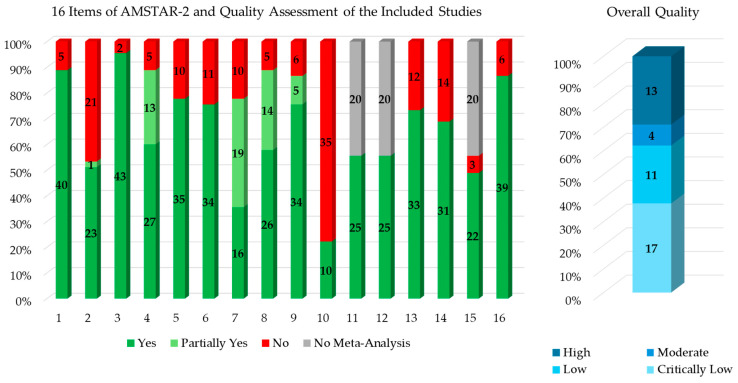
Graphical representation of the distribution of the 16 items of the AMSTAR-2 quality assessment and the overall quality judgment of the included studies.

Figure 4 summarizes the distribution of the 16 items of the AMSTAR-2 quality assessment and the overall quality judgment of the included studies.

The assessment of the primary studies overlap between the included systematic reviews and reveal a CCA of 2.2%, judged as “slight” according to Pieper et al. [25].

## 4. Discussion

The present systematic review of systematic reviews primarily aimed to assess the long-term effectiveness (≥6 months) and adherence of the different standalone digital tobacco cessation modalities in 164,010 current daily adult (≥18 years old) smokers of combustible tobacco, of which 142,069 were involved in an individual standalone digital tobacco cessation modality and 21,941 in a combined one.

A total of 45 systematic reviews [26,27,28,29,30,31,32,33,34,35,36,37,38,39,40,41,42,43,44,45,46,47,48,49,50,51,52,53,54,55,56,57,58,59,60,61,62,63,64,65,66,67,68,69,70] were included, comprising five different individual standalone digital tobacco cessation modalities: mobile text messaging (27,028), smartphone apps (18,448), Internet-based websites and programs (83,399), AI-based interventions (8055), and other digital tobacco modalities (5139), as well as eight different combinations of these interventions.

The related adherence and effectiveness were heterogeneously assessed using variations in both methodology (self-reported/biochemically verified by carbon monoxide tests or cotinine) and timing of CARs or PPA parameters. Therefore, the possibility to compare by a meta-analysis the adherence and effectiveness of the different subgroups of standalone digital tobacco cessation modalities was precluded due to the data heterogeneity.

### 4.1. Effectiveness

The WHO identified both the PPA and the CARs as long-term parameters crucial to decision-making, evaluated at least 6 months after the start of any smoking cessation interventions [7]. Smoking cessation effectiveness that was biochemically verified was recommended, if feasible, to improve the scientific rigor of clinical trials [71]. However, the tests needed for the biochemical verification have higher costs and need a rigorous follow-up plan to record measurements [71]. Indeed, smoking cessation effectiveness, as self-reported, was considered a valuable means in large-population-based clinical trials [71].

Regarding trustworthiness, in the present study, the smoking cessation effectiveness rates were higher when self-reported compared with those biochemically verified. Thus, in the interpretation of self-reported data only, the results of the previous meta-analysis by Patrick et al. [72] should be taken into account, which found that about 11% of subjects who self-report quitting smoking were not confirmed when verified biochemically.

#### 4.1.1. Effectiveness of Individual Standalone Digital Tobacco Cessation Modalities

**Figure 5:**
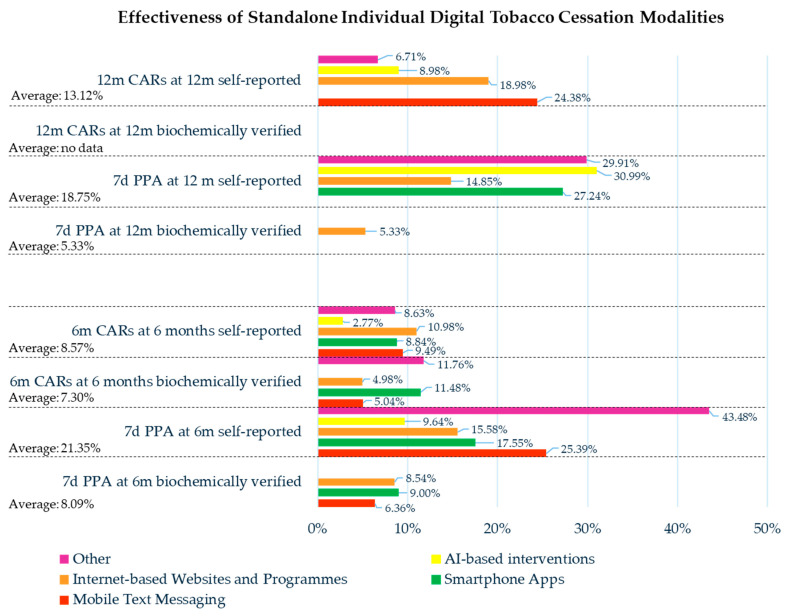
Effectiveness of standalone individual digital tobacco cessation modalities (both biochemically verified and self-reported CARs and 7-day PPA at 6 and 12 months).

The findings of the present umbrella reviews showed that the 6-month CARs at 6 months that were biochemically verified and the self-reported results of individual standalone digital tobacco cessation modalities had means of 7.30% and 8.57%, respectively; in particular, it was higher in the group categorized as “other” who used video materials or video counseling or telephone counseling (11.76%) and in the smartphone apps group (11.48%), while the effectiveness was markedly lower in the mobile text messaging group (5.04%) and in the Internet-based websites and programs group (4.98%) (Figure 5).

These findings showed that more interactive digital tools, such as video counseling or smartphone apps, were more effective compared with static formats [73]. The enhanced effectiveness, also confirmed by the PPA recorded rates at 6 months, was in accordance with a previous meta-analysis that highlighted the higher effectiveness of interactive and tailored digital smoking cessation programs [74].

Comparing these findings with the results of a previous umbrella review [8] that evaluated the effectiveness of digital support to pharmacological vs. non-pharmacological non-digital smoking cessation interventions, 6-month CARs at 6 months that were biochemically verified were slightly higher when digital tobacco modalities were used as a support for both pharmacological interventions (9.06%) and non-pharmacological non-digital interventions (14.85%) [8] than when digital modalities were used as an individual standalone digital smoking cessation intervention (7.30%).

These findings suggested that at 6 months, digital tobacco cessation modalities as a support slightly enhanced the intervention’s effectiveness compared with standalone digital tobacco programs, even those employing more interactive digital tools.

Unfortunately, none of the studies included reported the 12-month CARs at 12 months with biochemically verified rates, precluding the possibility of comparing these findings with the results of digital tobacco modalities used as a support. However, taking into account the 12-month CARs at 12 months self-reported, data showed a slightly higher rate when digital modalities were used as an individual standalone digital smoking cessation intervention (13.12%) than as a support for pharmacological or non-pharmacological interventions (11.79%) [8].

Of particular interest is the reversal of the reported effectiveness trend for standalone individual digital tobacco cessation modalities. In fact, compared with the 6-month trends, self-reported 12-month CARs data showed greater effectiveness for less interactive digital tools, such as Internet-based websites and programs (18.98%) and mobile text messaging (24.38%), compared with the “other” group (6.71%). These higher efficacy rates must be interpreted considering the self-reported assessment, which is not biochemically verified, but they still showed greater efficacy in the long term than more interactive programs.

This reversal may indicate that simplified interventions, such as mobile text messaging or web forms, may be more accepted by users over time because they fit more easily into the daily routine [75]. Thus, users may maintain engagement beyond the novelty period typical of more interactive tools [76]. The contrast between the 6-month results, which favor interactive digital tools, and the 12-month findings, in which less interactive digital tobacco modalities showed higher effectiveness, was in line with the existing literature. Simpler “push” digital tools, such as mobile text messaging and Internet-based websites, may maintain modest effectiveness for longer periods due to their ease of use and integration with daily life [75].

This pattern suggests that interactivity and personalization favor initial effectiveness, while simplicity and ease of integration with daily life favor long-term retention.

#### 4.1.2. Effectiveness of Individual vs. Combined Standalone Digital Tobacco Cessation Modalities

**Figure 6:**
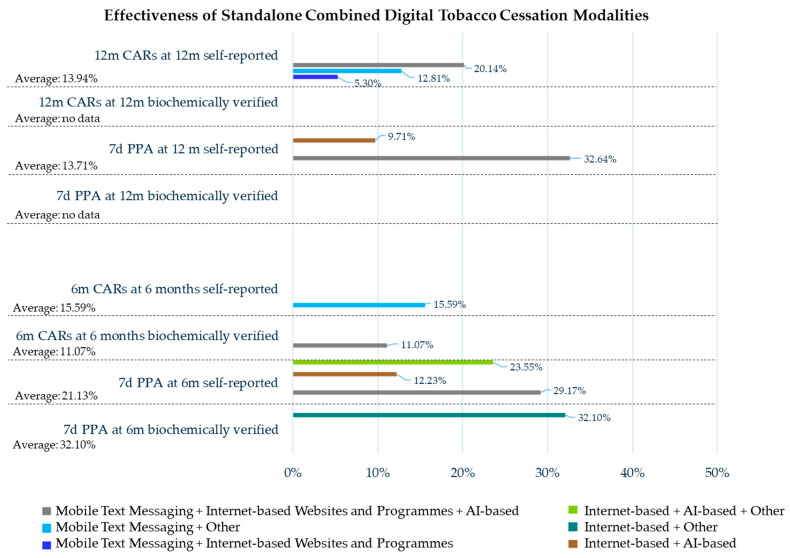
Effectiveness of standalone combined digital tobacco cessation modalities (both biochemically verified and self-reported CARs and 7-day PPA at 6 and 12 months).

The recorded effectiveness rates were highly heterogeneous in terms of both timing and methods of assessment (as shown in Table 3 and in Figure 6), so the comparison of effectiveness among the different and numerous associations of the standalone combined smoking cessation modalities was precluded.

However, comparing the average effectiveness of standalone individual or combined digital tobacco cessation modalities (as shown in Table 4), the standalone combined interventions showed higher effectiveness than the standalone individual interventions both at 6-month CARs at 6 months biochemically verified (11.07% vs. 7.30%, respectively) and 12-month CARs at 12 months self-reported (13.94% vs. 13.12%). Higher effectiveness was also reported for the PPA rates in the combined group.

The additive effectiveness of multi-component reinforcement through digital tobacco cessation modalities should be associated with a slight enhanced effectiveness, as also shown by Cantera et al. [77], who evaluated the effectiveness of multi-component smoking cessation interventions performed through two or more elements, including pharmacotherapy, non-pharmacological non-digital tools, and/or digital tools.

A key factor that should be considered when interpreting and comparing the effectiveness rates is the duration of the digital tobacco cessation modalities. Notably, combined interventions frequently extended over longer periods compared with individual modalities. In fact, combined treatments reported a minimum duration of 6 months (9.84% of cases), followed by 12 months (33.96%) and 54 weeks (5.91%). In contrast, individual interventions most commonly had a duration of 6 weeks (11.99%), 2 months (6.58%), and 6 months (9.90%).

This marked difference in intervention duration could be a contributor to the higher average effectiveness observed in combined digital tobacco cessation modalities, especially over the 6 months. Previous studies showed that intensive face-to-face smoking cessation interventions were also highly effective compared with shorter ones [78], and self-help interventions extended over the end of the smoking cessation intervention were associated with significantly higher abstinence rates [79]. A longer intervention duration may offer sustained support and reinforcement to smokers, solidifying their cessation commitment, overcoming relapses, and stabilizing behavioral changes over time [78,79].

Furthermore, considering the previous observation about the slightly greater effectiveness of highly interactive tools at 6 months and less interactive modalities at 12 months, the data suggest that interactivity drives early effectiveness, while simplicity and sustained engagement over a longer duration support longer-term effectiveness.

### 4.2. Adherence

Patients’ nonadherence is a challenge in clinical practice, which might result in lower effectiveness of the smoking cessation programs [80]. In the present study, the adherence rates were investigated based on the number of subjects who had finished the digital tobacco cessation interventions and were followed up, irrespective of their smoking status.

The present study found a higher average adherence rate (65.97%) at 6 months for standalone digital tobacco cessation modalities compared with the 47.73% mean adherence recorded in a previous umbrella review [8], which evaluated the adherence of digital tobacco cessation modalities used as a support for non-digital smoking cessation interventions. However, the authors also investigated the distinct adherence of digital support for pharmacological vs. non-pharmacological non-digital smoking cessation interventions, showing the highest adherence in digital supports to non-pharmacological interventions [8]. The proposed hypothesis was related to the early drop-out in the pharmacological group due to the early adverse effects of the drugs [8]. However, the higher 6-month adherence of digital support for non-pharmacological interventions compared with the present findings of standalone digital tobacco cessation modalities suggests that human interaction in the early stage should be a potent engagement driver.

At 12 months, the adherence recorded in the present study was 68.80% for standalone digital tobacco cessation modalities. In contrast, the previous umbrella review [8] reported higher adherence (77.62%) for digitally supported interventions. Notably, the group of digital support for pharmacological interventions registered the highest long-term adherence (83.92%) [8]. This change in trend was explained by considering that, despite the higher early drop-out rates due to the adverse effects of drugs, the long-term adherence was consistent.

These comparisons highlight the dynamic synergy between digital and non-digital tobacco cessation modalities. Both human-centered interactions and pharmacological therapy play crucial roles in shaping long-term adherence to digital tobacco cessation, as further detailed below when discussing the specific standalone digital modalities investigated.

#### 4.2.1. Adherence of Individual Standalone Digital Tobacco Cessation Modalities

The findings of the present study showed that at 6 months, the adherence rates of standalone individual digital tobacco cessation modalities had a mean of 65.84%; in particular, adherence was higher in the group categorized as “other” who used video materials or video counseling or telephone counseling (94.12%) and in the mobile text messaging group (83.76%), while adherence was markedly lower in the AI-driven group (42.5%).

These differences may have been the result of the varying levels of smoker engagement and perceived support across digital tobacco cessation modalities. Video and telephone counseling likely offer more tailored and interactive supports, which can enhance smokers’ motivation and accountability [5]. Similarly, mobile text messaging, even if less interactive, may provide consistent reinforcement and reminders, resulting in improved engagement [81]. In contrast, AI-driven interventions, such as email, quitline, and chatbot, while innovative, may lack the consistent or tailored reinforcement driven by healthcare providers that support adherence [82].

Confirming this, long-term adherence at 12 months in the AI-driven group (59.72%) was also lower than average (62.36%) and the lowest compared with the group categorized as “other” (64.08%) or the Internet-based websites and programs group (62.39%), although the difference showed a gradual leveling off.

These findings underscore the importance of considering the design of the digital tobacco cessation tools and the smoker’s experience when implementing digital strategies. While automation and scalability are key strengths of AI-driven tools, optimizing their design to improve adherence will be necessary to maximize their public health impact [83].

Instead, the observed gradual leveling off of adherence rates across digital tobacco cessation modalities at 12 months suggests that the smoker’s engagement may diminish over time, reflecting the expected decline in adherence commonly observed in long-term behavioral interventions [84]. It is conceivable that the initial advantage of more tailored and interactive supports of video or telephone counseling attenuates over time. Conversely, smokers who used less engaging digital tools, such as AI-driven groups, who remain in the smoking cessation program, may represent self-selected and motivated smokers, thereby stabilizing their adherence in the long term at 12 months. This observation is in line with previous theoretical models proposed by Yardley et al. (2016), which suggest that self-motivated users of digital tools for health interventions may adhere to the programs even with less interactive digital tools [85].

#### 4.2.2. Adherence of Individual vs. Combined Standalone Digital Tobacco Cessation Modalities

**Figure 7:**
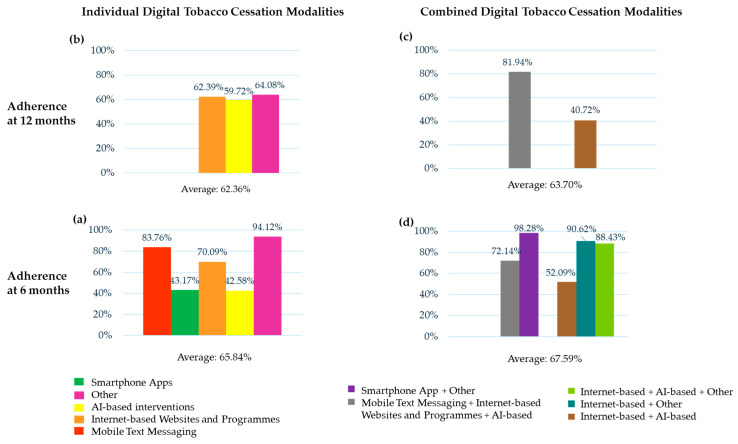
Adherence of standalone digital tobacco cessation modalities: (**a**) individual at 6 months; (**b**) individual at 12 months; (**c**) combined at 12 months; (**d**) combined at 6 months. At 6 months, the combination of smartphone app and “other” modalities resulted in remarkably high adherence (98.28%) consistently from 2 weeks to 6 months, which was higher than any individual standalone digital tobacco modalities, including the “other” group alone (94.12%). Similarly, the Internet-based with “other” (90.6%) and also with AI-driven tools (88.43%) at 6 months reinforced the adherence of their respective individual group (70.9% for the Internet-based and programs group and 42.5% for the AI-driven ones). These findings reinforce the importance of human-centered interaction, which can be obtained using “other” digital tools like video or telephone counseling. Furthermore, these results suggest that integrating less engaging digital tools, like AI-driven modalities with more structured tools or smartphone apps or Internet-based websites and programs, may mitigate the adherence challenges associated with AI-only driven interventions and may also enhance the adherence of structured digital tools.

The recorded adherence rates showed that combining standalone digital tobacco cessation modalities often yielded higher retention rates compared with individual standalone ones, although the adherence rates appear to vary in relation to the specific combination used (Figure 7).

Interestingly, long-term adherence at 12 months was relatively high at 81.94% in the mobile text messaging with Internet-based websites and programs and AI-driven tools group, notably higher than any long-term adherence rates achieved by any individual standalone digital tobacco cessation modalities. This result suggests that multi-component interventions may improve motivation and adherence over time, limiting the expected drop-out observed in long-term behavioral interventions.

Overall, these findings underscore that individual standalone digital tobacco cessation modalities involving human-centered interactions can obtain high adherence even when combined with multiple digital tobacco cessation modalities. These findings are consistent with the “Supportive Accountability Model” proposed by Mohr et al. (2011) [86], which suggested that combining multiple digital modalities, particularly when at least one comprises a human-centered interaction, can significantly enhance adherence. In line with this model [86], the high adherence rates registered in combinations involving video or telephone counseling may be related to the perceived social support and personalization by smokers.

However, the choice of which digital tobacco modalities should be combined is crucial because not all combinations showed beneficial effects on adherence to digital tobacco cessation modalities (such as the addition of AI-driven tools to the Internet websites and programs).

### 4.3. Relationship Between Effectiveness and Adherence of Digital Tobacco Cessation Modalities

The trends of effectiveness and adherence, as presented in Figure 5, Figure 6 and Figure 7 for standalone individual and combined digital tobacco cessation modalities, reveal a complex and bidirectional relationship.

Conventionally, higher adherence to an intervention is expected to lead to higher effectiveness rates [87]. As shown in Figure 7, standalone combined digital tobacco cessation modalities exhibited higher average adherence rates (Figure 7c,d) and also showed superior average effectiveness compared with individual modalities (Figure 5 and Figure 6). This relationship is also highlighted by comparing the effectiveness and adherence relationship of specific combined modalities. For example, this direct relationship is particularly evident when comparing the higher average adherence at 12 months of mobile text messaging plus Internet-based websites and programs plus AI-based interventions (81.94%) vs. the lower adherence of the Internet-based plus AI-based interventions (40.72%). The same trends are reflected in the effectiveness at 7-day PPA at 12 months when self-reported, which were 32.64% and 9.71%, respectively (Figure 6 and Figure 7).

However, the bidirectional relationship between effectiveness and adherence in digital tobacco cessation is not linear in all cases. For example, while mobile text messaging achieved higher adherence (83.76%) at 6 months compared with the average adherence of individual digital tobacco modalities, it yielded lower biochemically verified effectiveness (both 6.36% 7-day PPA and 5.04% 6-month CARs) compared with the registered average. These divergence findings demonstrate that high adherence does not always translate directly into higher effectiveness [88].

On the one hand, these findings should be interpreted considering adherence as a necessary but not sufficient key for achieving high effectiveness [89]. Taking into account the aforementioned example, mobile text messaging, despite strong engagement as reminders that sustain participation, lacks the therapeutic depth required to change smokers’ behavioral smoking status in the early intervention phase [89].

On the other hand, the relationship between adherence and effectiveness is not strictly unidirectional [8,10,11]. It is possible that smokers who achieve early success become more motivated to continue to adhere to the intervention, while those who experience a relapse may not continue to adhere to the intervention [8,10,11]. The high 6-month adherence rate observed in the mobile text messaging group, despite its low effectiveness, may reflect this dynamic: continued mobile text messaging adherence likely indicates that adherent smokers were self-motivated to remain engaged, whereas those who experienced relapses may drop out. Thus, adherence may be a key to effectiveness as much as a cause. This complex, bidirectional dynamic means that successful smokers reinforce their adherence, while relapse can accelerate disengagement [8,10,11].

Notably, to overcome the limitations of digital tobacco cessation modalities, which often maintain high adherence through reminders but lack therapeutic depth, combined intervention strategies that employ highly interactive and human-centered digital tools during early intervention phases, followed by simpler, low-burden modalities for long-term support, may offer a reliable solution to optimize both adherence and effectiveness.

### 4.4. Limitations, Unaddressed Knowledge Gaps, and Future Directions

Some limitations should be considered when interpreting the results of the present systematic review of systematic reviews. In fact, the AMSTAR-2 tool revealed that despite about 40% of the included systematic reviews being rated as high or moderate quality, the remaining 60% were rated as low or critically low quality. This suggests that potential biases may undermine the robustness of the conclusions. In particular, the most frequent methodological weaknesses observed in the included systematic reviews are in order as follows (as shown in Figure 4):

- The lack of funding sources for the primary studies, which may undermine the assessment of potential conflicts of interest;
- The absence of a registered protocol, which raises concerns about potential reporting biases;
- The failure to discuss the heterogeneity observed in the results, which may potentially mislead the synthesis of the evidence.

Conversely, the most frequent strengths of the included studies are in order as follows (as shown in Figure 4):

- The clear explanations of the study design selection, which enhance transparency;
- The adherence to the PICO models and the establishment of a research question, which ensures a rigorous methodological approach to evidence synthesis and facilitates reproducibility;
- The clarification about any potential sources of conflict of interest, which contributes to the trustworthiness of the findings.

In the present study, the absence of standardized follow-up intervals introduced heterogeneity in the assessment of digital tobacco cessation effectiveness at each time point. This variability allowed for only a qualitative synthesis of the data, limiting the ability to perform parallel comparisons among the different subtypes of standalone digital tobacco cessation modalities. Moreover, the methodological heterogeneity in effectiveness assessment methods—both PPA and CARs were reported as self-reported or biochemically validated measures at different time points—further hindered the possibility of conducting a quantitative analysis of the results.

Despite this heterogeneity and the numerous different types of interventions found, some combinations between the different digital tobacco cessation modalities have never been reported. Exploring additional combinations (such as smartphone apps plus Internet-based websites and programs) could offer more personalized and engaging approaches to cessation programs.

Another gap in the current findings is the underrepresentation of older populations. The average age of participants currently being investigated among studies is relatively young (from a mean of 20.42 to 55.87 years old), highlighting the need for further research to compare the effectiveness, adherence, satisfaction, and usability of digital tobacco cessation modalities among older adults and young adults. A previous study showed that older adults engage with digital tobacco cessation modalities at rates comparable with younger adults and exhibit similar quit rates, suggesting that digital tools can be effective across age groups [90]. However, the specific effectiveness, adherence, satisfaction, and usability of the different digital tobacco cessation modalities among older adults remain insufficiently explored. Given the potential barriers to technology use in older adults, it is crucial to conduct further targeted studies [91].

Based on the same goal, future directions should prioritize the assessment of the acceptability. Indeed, no data concerning acceptability were retrieved in the present study. However, it might be conceivable that lower acceptability rates could potentially decrease the effectiveness and adherence of the interventions. Thus, understanding user acceptance should be crucial for refining and implementing digital tobacco cessation modalities.

Finally, the current data available, unfortunately, did not allow the investigation of our secondary aim, which was established before starting the research during the study protocol drafting: the assessment of the effect of digital tobacco cessation modalities on smokers’ health.

Investigating the long-term health benefits—encompassing medical (cardiovascular/pneumological/metabolic/psychological) and oral (periodontal/peri-implant/mucosal lesions) parameters—is essential for a comprehensive assessment of the impact of digital tobacco cessation modalities on overall health and well-being. This approach will address existing knowledge gaps regarding the effects of smoking cessation on human health, particularly concerning periodontal diseases [92].

### 4.5. Strengths

To the best of our current knowledge and evidence, the present systematic review of systematic reviews pioneered in evaluating and comparing the long-term effectiveness (≥6 months) and adherence of the different individual and combined standalone digital tobacco cessation modalities. Including 164,010 current daily adult (≥18 years old) smokers of combustible tobacco, as well as large samples of each intervention analyzed, enhanced the generalizability of the findings, ensuring a comprehensive representation of the intended population, thus reinforcing the reliability of the conclusion. In addition, it should be considered that despite the large samples of each intervention analyzed, several potential confounders were excluded by the eligibility criteria, such as non-daily smokers of combustible tobacco, younger subjects (<18 years old), smokers drinking alcohol or with disorders of substance abuse, and pregnant and lactating women. This methodology guaranteed that the presented findings focused on a well-defined target population, thereby minimizing the influence of potential population confounding.

Furthermore, even if the overlap of primary studies across systematic reviews is a recognized potential source of bias in umbrella review studies [25], in the present study, the overlap was judged as “slight” (CCA = 2.2%). This finding suggested that the risk of redundancy among the included evidence was minimal and that the strength of the conclusions was unlikely to have been affected by overlapping data.

Another point of strength lies in its focus on timely and relevant topics, underpinned by evidence from the recent WHO guidelines (2024) [7], intending to evaluate aspects that remain underexplored in the existing literature. As a consequence, even if no restrictions on the study’s years of publication were applied in the search strategy, all the included systematic reviews [26,27,28,29,30,31,32,33,34,35,36,37,38,39,40,41,42,43,44,45,46,47,48,49,50,51,52,53,54,55,56,57,58,59,60,61,62,63,64,65,66,67,68,69,70] were relatively recently published in the last 16 years (2009–2024). The high number of systematic reviews on digital tobacco cessation modalities in the last 16 years highlights the widespread role of digital tools and reflects the growing interest in developing new telemedicine frontiers for public health goals [93].

## 5. Conclusions

The present systematic review of systematic reviews provides a comprehensive evaluation of the long-term effectiveness and adherence of standalone individual and combined administered digital tobacco cessation modalities, analyzing data from 45 systematic reviews and including over 164,010 adult daily smokers of combustible tobacco.

At 6 months, highly interactive and human-centered digital tools, such as smartphone apps and video or telephone counseling, registered higher effectiveness (CARs biochemically verified were 11.48% and 11.76%, respectively).

In contrast, simpler, less interactive digital tools like mobile text messaging and Internet-based programs showed higher effectiveness at 12 months (self-reported CARs were 24.38% and 18.98%, respectively). These digital smoking cessation modalities were the most frequently investigated (in 27,028 and 83,399 smokers, respectively), and the related findings suggest that initial engagement may be driven by interactive digital tools, while long-term effectiveness is more closely associated with interventions that provide sustained support but are easy to integrate into daily life. In fact, at 12 months, compared with individually delivered interventions, combined digital tobacco cessation modalities demonstrated slightly higher effectiveness (self-reported CARs were 13.12% vs. 13.94%) and adherence (62.36% vs. 63.70%). This enhanced performance can be largely attributed to the sustained support provided by multi-component approaches and their longer smoking cessation intervention durations. However, even with the larger sample size of combined digital tobacco cessation modalities (21,941), it should be noted that the individual ones registered a much larger sample (142,069) with a stronger force of evidence.

Adherence to digital tobacco cessation modalities is generally high, particularly when human-centered digital tools are involved, amounting to 94.12% at 6 months and 64.08% at 12 months. Video or telephone counseling, even when integrated with less engaging tools, like AI-driven interventions, demonstrates higher adherence rates. These findings underscore the crucial role of perceived social support and digital tools’ personalization in maintaining users’ engagement.

Despite these advances, the field faces significant challenges. The certainty of evidence remains limited by the quality of the included systematic review. In fact, 60% of them were rated as low or critically low quality. Another limitation that undermines the strength of evidence of the present conclusion account is the heterogeneity of the results of the included studies, including in assessment methodologies and a notable lack of biochemically verified 12-month abstinence rates. Furthermore, there is an urgent need for research addressing older populations, evaluating user acceptability, and investigating the long-term health benefits of digital cessation interventions.

From a clinical perspective, the strongest evidence supports prioritizing multi-component digital tobacco cessation interventions that incorporate human-centered interactions for initial engagement, alongside simpler, sustained digital support to enhance long-term effectiveness and adherence.

## Figures and Tables

**Table 1:** Search strategy: database, last date of search, advanced search string, and filters.

Database	Date of Search	Search String	Filters
MEDLINE/ PubMed	10 Oct 2024	((“smoking cessation”[All Fields] OR “stopping smoking”[All Fields] OR “quitting smoking”[All Fields] OR “ex-smokers”[All Fields] OR “giving up smoking”[All Fields]) AND (“cell-phone”[All Fields] OR “text messaging”[All Fields] OR (“smartphone”[MeSH Terms] OR “smartphone”[All Fields] OR “smartphones”[All Fields] OR “smartphone s”[All Fields]) OR “social media”[All Fields] OR “computers”[All Fields] OR “online systems”[All Fields] OR “computer handheld”[All Fields] OR ((“mobile”[All Fields] OR “mobiles”[All Fields]) AND “appli cations”[All Fields]) OR “technology”[All Fields] OR “virtual reality”[All Fields] OR (“telemedicine”[MeSH Terms] OR “telemedicine”[All Fields] OR “telemedicine s”[All Fields]) OR “augmented reality”[All Fields] OR (“multimedia”[MeSH Terms] OR “multimedia”[All Fields] OR “multimedium”[All Fields]) OR “internet-based intervention”[All Fields] OR “electronic mail”[All Fields]) AND “systematic review”[All Fields]) AND ((systematicreview[Filter]) AND (english[Filter]))	Systematic Review; English
Scopus	10 Oct 2024	TITLE-ABS-KEY ((“smoking cessation” OR “stopping smoking” OR “quitting smoking” OR “ex-smokers” OR “giving up smoking”) AND (“cell-phone” OR “text messaging” OR smartphone OR “social media” OR “computers” OR “online systems” OR “computer handheld” OR “mobile appli-cations” OR “technology” OR “virtual reality” OR telemedicine OR “augmented reality” OR multimedia OR “internet-based intervention” OR “electronic mail”) AND (“systematic review”)) AND (LIMIT-TO (DOCTYPE, “re”)) AND (LIMIT-TO (LANGUAGE, “English”))	Review; English
Web of Science	10 Oct 2024	(“smoking cessation” OR “stopping smoking” OR “quitting smoking” OR “ex-smokers” OR “giving up smoking”) AND (“cell-phone” OR “text messaging” OR smartphone OR “social media” OR “computers” OR “online systems” OR “computer handheld” OR “mobile appli-cations” OR “technology” OR “virtual reality” OR telemedicine OR “augmented reality” OR multimedia OR “internet-based intervention” OR “electronic mail”) AND (“systematic review”) (All Fields) and Review Article (Document Types) and English (Languages)	Review; English
PROSPERO	30 Dec 2024	(“smoking cessation” OR “stopping smoking” OR “quitting smoking” OR “ex-smokers” OR “giving up smoking”) AND (“cell-phone” OR “text messaging” OR smartphone OR “social media” OR “computers” OR “online systems” OR “computer handheld” OR “mobile appli-cations” OR “technology” OR “virtual reality” OR telemedicine OR “augmented reality” OR multimedia OR “internet-based intervention” OR “electronic mail”) AND (“systematic review”) (All Fields) and English (Languages)	English

**Table 2:** Individual digital tobacco cessation modalities. Outcomes are extracted and clustered for each individual digital tobacco cessation modality (mobile text messaging, smartphone apps, Internet-based and websites and programs, AI-based interventions, other digital tobacco modalities): effectiveness (CARs and PPA, biochemically verified and self-reported, sorted in chronological order); adherence (sorted in chronological order); satisfaction.

	Mobile Text Messaging	Smartphone Apps	Internet-Based Websites and Programs	AI-Based Interventions	Other Digital Tobacco Modalities
Effectiveness: CARs Former Smokers/Smokers Assessed (Former Smoker %)					
For 3 m at 6 m Self-reported	—	465/1798 (25.86%)	—	—	—
For 3 m at 6 m N/D methods	—	—	—	—	24/430 (5.58%)
For 6 m at 6 m Biochemically verified	138/2738 (5.04%)	225/1960 (11.48%)	48/964 (4.98%)	—	6/51 (11.76%)
For 6 m at 6 m Self-reported	237/2738 (9.49%)	890/10,070 (8.84%)	388/3534 (10.98%)	55/1982 (2.77%)	78/904 (8.63%)
For 6 m at 7 m Biochemically verified	—	—	1828/18,452 (9.91%)	—	—
For 6 m at 7 m Self-reported	104/1688 (6.16%)	—	552/6256 (8.82%)	—	—
For 6.5 m at 6.5 m Self-reported	—	104/850 (12.24%)	—	—	—
For 6 m at 12 m Self-reported	—	—	—	14/163 (8.59%)	—
For 12 m at 12 m Self-reported	78/320 (24.38%)	—	703/3703 (18.98%)	139/1548 (8.98%)	198/2952 (6.71%)
For 18 m at 18 m Self-reported	—	—	159/3990 (3.98%)	—	—
For 24 m at 24 m Self-reported	—	—	234/1926 (12.15%)	—	—
Effectiveness: PPA Former Smokers/Smokers Assessed (Former Smoker %)					
7 d PPA at 6 m Biochemically verified	96/1509 (6.36%)	144/1600 (9.00%)	218/2554 (8.54%)	—	—
7 d PPA at 6 m Self-reported	5135/20,226 (25.39%)	1308/7452 (17.55%)	697/4473 (15.58%)	236/2447 (9.64%)	20/46 (43.48%)
30 d PPA at 6 m Biochemically verified	—	—	75/420 (17.86%)	—	—
30 d PPA at 6 m Self-reported	58/317 (18.30%)	504/4920 (10.24%)	318/2427 (13.10%)	265/814 (32.55%)	436/1727 (25.25%)
N/D time PPA at 6 m Biochemically verified	—	—	108/964 (11.20%)	—	—
N/D time PPA at 6 m Self-reported	—	—	2792/18,686 (14.94%)	—	—
N/D methods and time at 6 m	—	—	—	27/205 (13.17%)	54/80 (67.50%)
7 d PPA at 6.5 m Self-reported	—	134/850 (15.76%)	—	—	—
7 d PPA at 7 m Self-reported	340/1688 (20.14%)	—	1527/5581 (27.36%)	—	—
30 d PPA at 7 m Biochemically verified	—	—	308/1820 (16.92%)	—	—
30 d PPA at 7 m Self-reported	—	—	420/1820 (23.08%)	—	—
N/D methods and time at 7 m	—	—	17/65 (26.15%)	—	—
30 d PPA at 9 m Self-reported	—	—	71/307 (23.13%)	—	—
7 d PPA at 11.5 m Self-reported	—	—	102/272 (37.50%)	—	—
30 d PPA at 11.5 m Self-reported	—	—	183/1686 (10.85%)	—	—
7 d PPA at 12 m Biochemically verified	—	—	84/1577 (5.33%)	—	—
7 d PPA at 12 m Self-reported	—	1316/4830 (27.24%)	2103/14,160 (14.85%)	181/584 (30.99%)	189/632 (29.91%)
30 d PPA at 12 m Biochemically verified	—	—	357/1953 (18.28%)	—	22/171 (12.87%)
30 d PPA at 12 m Self-reported	—	—	874/4674 (18.70%)	—	168/757 (22.19%)
N/D time PPA at 12 m Biochemically verified	—	—	24/952 (2.52%)	—	—
N/D time PPA at 12 m Self-reported	—	—	56/952 (5.88%)	—	—
N/D methods and time at 12 m	—	—	14/312 (4.49%)	—	—
30 d PPA at 13 m Self-reported	—	—	1436/12,904 (11.13%)	—	—
N/D methods and time at 13 m	—	—	594/5404 (10.99%)	—	—
30 d PPA at 18 m Self-reported	—	—	726/3990 (18.20%)	—	—
7 d PPA at 24 m Biochemically verified	—	—	255/1926 (13.24%)	—	—
Adherence					
At 6 m	9312/11,118 (83.76%)	5809/13,456 (43.17%)	18,995/27,101 (70.09%)	195/458 (42.58%)	48/51 (94.12%)
At 7 m	1424/1688 (84.36%)	—	7144/8076 (88.46%)	—	—
At 11.5 m	—	—	816/1686 (48.40%)	—	—
At 12 m	—	—	5719/9167 (62.39%)	943/1579 (59.72%)	1438/2244 (64.08%)
At 18 m	—	—	2745/3990 (68.80%)	—	—
Satisfaction					
At 6 m	256/320 (80.00%) satisfied or totally satisfied	—	8.59 mean of the Perceived Usefulness and Ease of Use Scale in 486 subjects with schizophrenia	—	—

Acronyms: percentage (%); no data available (—); continuous abstinence rates (CARs); point prevalence abstinence (PPA). In the “Effectiveness” section, the lines marked in grey highlight the biochemically assessed effectiveness rates, those in white the self-report, and for those in light blue, the method was not specified.

**Table 3:** Combined digital tobacco cessation modalities. Outcomes are extracted and clustered for each combined digital tobacco cessation modality (every combination between mobile text messaging and/or smartphone apps and/or Internet-based and/or websites and programs and/or AI-based interventions and/or other digital tobacco modalities): effectiveness (CARs and PPA, biochemically verified and self-reported, sorted in chronological order); adherence (sorted in chronological order).

	Mobile Text Messaging + Internet-Based	Mobile Text Messaging + Other	Mobile Text Messaging + Internet-Based + AI-Based	Smartphone App + Other	AI-Based + Other	Internet-Based + AI-Based	Internet-Based + Other	Internet-Based + AI-Based Other
Effectiveness: CARs Former Smokers/Smokers Assessed (Former Smoker %)								
For 3 m at 6 m Self-reported	—	—	—	—	—	56/877 (6.39%)	—	—
For 4 m at 6 m Biochemically verified	—	—	—	43/58 (74.14%)	—	—	—	—
For 4.6 m at 6 m Self-reported	—	—	—	—	—	46/1104 (4.16%)	—	—
For 6 m at 6 m Biochemically verified	—	—	116/1048 (11.07%)	—	—	—	—	—
For 6 m at 6 m Self-reported	—	566/3631 (15.59%)	—	—	—	—	—	—
For 9 m at 9 Self-reported	—	523/3631 (14.40%)	—	—	—	—	—	—
For 12 m at 12 m Self-reported	24/453 (5.30%)	465/3631 (12.81%)	261/1296 (20.14%)	—	—	—	—	—
Effectiveness: PPA Former Smokers/Smokers Assessed (Former Smoker %)								
7 d PPA at 6 m Biochemically verified	—	—	—	—	—	—	130/405 (32.10%)	—
7 d PPA at 6 m Self-reported	—	—	378/1296 (29.17%)	—	—	167/1366 (12.23%)	—	171/726 (23.55%)
30 d PPA at 6 m Biochemically verified	—	—	—	—	—	—	90/405 (22.22%)	—
30 d PPA at 6 m Self-reported	451/2570 (17.55%)	—	—	—	—	—	—	—
N/D time PPA at 6 m Biochemically verified	—	—	164/1048 (15.65%)	—	—	—	—	—
7 d PPA at 7 m Self-reported	—	—	150/509 (29.47%)	—	—	1064/1799 (59.14%)	—	—
30 d PPA at 7 m Biochemically verified	—	—	—	—	—	595/1799 (33.07%)	—	—
30 d PPA at 7 m Self-reported	—	—	—	—	—	728/1799 (40.47%)	—	—
30 d PPA at 9 m Self-reported	72/311 (23.15%)	—	—	—	—	—	—	—
1 d PPA at 12 m Self-reported	—	—	—	—	18/213 (8.45%)	—	—	—
7 d PPA at 12 m Self-reported	—	—	423/1296 (32.64%)	—	—	595/6130 (9.71%)	—	—
30 d PPA at 12 m Biochemically verified	—	13/221 (5.88%)	—	—	—	—	—	—
30 d PPA at 12 m Self-reported	569/2570 (22.14%)	90/221 (40.72%)	—	—	—	—	—	—
N/D methods and time at 12 m	—	—	—	—	22/328 (6.71%)	—	—	—
Adherence								
At 2 w	—	—	—	57/58 (98.28%)	—	—	—	—
At 1 m	—	—	—	57/58 (98.28%)	—	—	—	—
At 2 m	—	—	—	57/58 (98.28%)	—	—	—	—
At 3 m	—	—	—	57/58 (98.28%)	—	—	—	—
At 6 m	—	—	756/1048 (72.14%)	57/58 (98.28%)	—	1032/1981 (52.09%)	367/405 (90.62%)	639/726 (88.43%)
At 7 m	—	—	—	—	—	1673/1799 (93.00%)	—	—
At 12 m	—	—	1062/1296 (81.94%)	—	—	419/1029 (40.72%)	—	—

Acronyms: percentage (%); no data available (—); continuous abstinence rates (CARs); point prevalence abstinence (PPA). In the “Effectiveness” section, the lines marked in grey highlight the biochemically assessed effectiveness rates, those in white the self-report, and in those in light blue, the method was not specified.

**Table 4:** Individual vs. combined digital tobacco cessation modalities and overall. Outcomes are clustered for all individual vs. combined digital tobacco cessation modalities and overall.

	Individual Overall	Combined Overall	Overall (Individual Plus Combined)
Effectiveness: CARs			
For 3 m at 6 m Self-reported	465/1798 (25.86%)	56/877 (6.39%)	521/2675 (19.48%)
N/D methods for 3 m at 6 m	24/430 (5.58%)	—	24/430 (5.58%)
For 4 m at 6 m Biochemically verified	—	43/58 (74.14%)	43/58 (74.14%)
For 4.6 m at 6 m Self-reported	—	46/1104 (4.16%)	46/1104 (4.16%)
For 6 m at 6 m Biochemically verified	417/5713 (7.30%)	116/1048 (11.07%)	533/6761 (7.88%)
For 6 m at 6 m Self-reported	1648/19,228 (8.57%)	566/3631 (15.59%)	2214/22,859 (9.69%)
For 6 m at 7 m Biochemically verified	1828/18,452 (9.91%)	—	1828/18,452 (9.91%)
For 6 m at 7 m Self-reported	656/7944 (8.26%)	—	656/7944 (8.26%)
For 6.5 m at 6.5 m Self-reported	104/850 (12.24%)	—	104/850 (12.24%)
For 9 m at 9 m Self-reported	—	523/3631 (14.40%)	523/3631 (14.40%)
For 6 m at 12 m Self-reported	14/163 (8.59%)	—	14/163 (8.59%)
For 12 m at 12 m Self-reported	1118/8523 (13.12%)	750/5380 (13.94%)	1868/13,903 (13.44%)
For 18 m at 18 m Self-reported	159/3990 (3.98%)	—	159/3990 (3.98%)
For 24 m at 24 m Self-reported	234/1926 (12.15%)	—	234/1926 (12.15%)
Effectiveness: PPA			
7 d PPA at 6 m Biochemically verified	458/5663 (8.09%)	130/405 (32.10%)	588/6068 (9.69%)
7 d PPA at 6 m Self-reported	7396/34,644 (21.35%)	716/3388 (21.13%)	8112/38,032 (21.33%)
30 d PPA at 6 m Biochemically verified	75/420 (17.86%)	90/405 (22.22%)	165/825 (20.00%)
30 d PPA at 6 m Self-reported	1581/10,205 (15.49%)	451/2570 (17.55%)	2032/12,775 (15.91%)
N/D time PPA at 6 m Biochemically verified	108/964 (11.20%)	164/1048 (15.65%)	272/2012 (13.52%)
N/D time PPA at 6 m Self-reported	2792/18,686 (14.94%)	—	2792/18,686 (14.94%)
N/D methods and time at 6 m	81/285 (28.42%)	—	81/285 (28.42%)
7 d PPA at 6.5 m Self-reported	134/850 (15.76%)	—	134/850 (15.76%)
7 d PPA at 7 m Self-reported	1867/7269 (25.68%)	1214/2308 (52.73%)	3081/9577 (32.17%)
30 d PPA at 7 m Biochemically verified	308/1820 (16.92%)	595/1799 (33.07%)	903/3619 (24.95%)
30 d PPA at 7 m Self-reported	420/1820 (23.08%)	728/1799 (40.47%)	1148/3619 (31.72%)
N/D methods and time at 7 m	17/65 (26.15%)	—	17/65 (26.15%)
30 d PPA at 9 m Self-reported	71/307 (23.13%)	72/311 (23.15%)	143/618 (23.14%)
7 d PPA at 11.5 m Self-reported	102/272 (37.50%)	—	102/272 (37.50%)
30 d PPA at 11.5 m Self-reported	183/1686 (10.85%)	—	183/1686 (10.85%)
1 d PPA at 12 m Self-reported	—	18/213 (8.45%)	18/213 (8.45%)
7 d PPA at 12 m Biochemically verified	84/1577 (5.33%)	—	84/1577 (5.33%)
7 d PPA at 12 m Self-reported	3789/20,206 (18.75%)	1018/7426 (13.71%)	4807/27,632 (17.40%)
30 d PPA at 12 m Biochemically verified	379/2124 (17.84%)	13/221 (5.88%)	392/2345 (16.72%)
30 d PPA at 12 m Self-reported	1042/5431 (19.19%)	659/2791 (23.61%)	1701/8222 (20.69%)
N/D time PPA at 12 m Biochemically verified	24/952 (2.52%)	—	24/952 (2.52%)
N/D time PPA at 12 m Self-reported	56/952 (5.88%)	—	56/952 (5.88%)
N/D methods and time at 12 m	14/312 (4.49%)	22/328 (6.71%)	36/640 (5.63%)
30 d PPA at 13 m Self-reported	1436/12,904 (11.13%)	—	1436/12,904 (11.13%)
N/D methods and time at 13 m	594/5404 (10.99%)	—	594/5404 (10.99%)
30 d PPA at 18 m Self-reported	726/3990 (18.20%)	—	726/3990 (18.20%)
7 d PPA at 24 m Biochemically verified	255/1926 (13.24%)	—	255/1926 (13.24%)
Adherence			
At 2 w	—	57/58 (98.28%)	57/58 (98.28%)
At 1 m	—	57/58 (98.28%)	57/58 (98.28%)
At 2 m	—	57/58 (98.28%)	57/58 (98.28%)
At 3 m	—	57/58 (98.28%)	57/58 (98.28%)
At 6 m	34,359/52,184 (65.84%)	2851/4218 (67.59%)	37,210/56,402 (65.97%)
At 7 m	8568/9764 (87.75%)	1673/1799 (93.00%)	10,241/11,563 (88.57%)
At 11.5 m	816/1686 (48.40%)	—	816/1686 (48.40%)
At 12 m	8100/12,990 (62.36%)	1481/2325 (63.70%)	9581/15,315 (62.56%)
At 18 m	2745/3990 (68.80%)	—	2745/3990 (68.80%)
Satisfaction			
At 6 m	256/320 (80.00%) satisfied or totally satisfied; 8.59 mean of the Perceived Usefulness and Ease of Use Scale in 486 subjects with schizophrenia	—	256/320 (80.00%) satisfied or totally satisfied; 8.59 mean of the Perceived Usefulness and Ease of Use Scale in 486 subjects with schizophrenia

Acronyms: percentage (%); no data available (—); continuous abstinence rates (CARs); point prevalence abstinence (PPA). In the “Effectiveness” section, the lines marked in grey highlight the biochemically assessed effectiveness rates, those in white the self-report, and in those in light blue, the method was not specified.

## Data Availability

Data are available in the MEDLINE/PubMed, BioMed, Web of Science, and Scopus databases and in the PROSPERO register.

## Supplementary Materials

The following supporting information can be downloaded at: https://www.mdpi.com/article/10.3390/healthcare13172125/s1, File S1: Table of data extraction; File S2: Digital tobacco cessation modalities characteristics; File S3: Subgroups of the digital tobacco cessation modalities; File S4: Quality assessment.
