# Supplementary material for: Effectiveness and Adherence of Standalone Digital Tobacco Cessation Modalities: A Systematic Review of Systematic Reviews
Source: Healthcare (Basel). 2025 Aug 26;13(17):2125. doi: 10.3390/healthcare13172125 (PMC12428045; doi:10.3390/healthcare13172125)
Supplement: Supplementary file 1 [file healthcare-13-02125-s001.zip › 18.05.25 Supplementary File S2.pdf]

Supplementary File S2: Digital Tobacco Cessation Modalities

Table S1 displays the characteristics of digital tobacco cessation modalities reported by each alphabetically ordered included systematic review.

Table S1. Digital tobacco cessation modalities: sample size, digital tobacco cessation modalities type, and characteristics (frequency and duration).
Abbreviations: number (n.); Technology-based Care (TC); Missing Data (MD); Not Derivable (N/D); Interactive Voice Response, "IVR".

Study Features	Sample size and Digital Tobacco Cessation Modalities		Frequency	Duration
Barnett A., 2020 [63]	n.143 TC	Mobile phone App (SmartQuit)	MD	22 days
	n.182 TC	Mobile phone App (QuitGuide)	MD	22 days
Barroso-Hurtado M., 2021 [26]	n.820 TC	Mobile phone App (Crush the Crave)	MD	MD
	n.342 TC	Mobile phone App with mandatory information about quitting options, daily motivational push notifications, quitting diary, and a quitting tracker	MD	MD
	n.342 TC	Mobile phone App with non-mandatory information about quitting options	MD	MD
	n.143 TC	Mobile phone App (SmartQuit)	MD	22 days
	n.182 TC	Mobile phone App (QuitGuide)	MD	22 days
Bendotti H., 2023 [27]	n.205 TC	Chatbot groups (WeChat)	MD	MD
Boland C.V., 2018 [28]	n.852 TC	Tailored mobile phone text-message	5 text-message/days for the first 6 weeks<br>3 text-message/days for other 20 weeks	26 weeks
	n.853 TC	Mobile phone text-message	1 text-message/15 days	26 weeks
	n.2321 TC	Interactive tailored website resources	MD	>2 months
	n.2292 TC	Static one-page website resources	MD	MD
Brown J., 2013	n.260 TC	Standard government website resources (QuitNet.com)	MD	MD
	n.257	Website-tailored (RealU Website) resources	MD	20 weeks

[64]	TC	Email		30 weeks
Byambasuren O.,<br>2023<br><br>[29]	n.229<br>TC	Video counseling delivered via video community technology (Skype)	Up to 6 counseling of 15 minutes each	MD
	n.201<br>TC	Telephone counseling	Up to 6 counseling 15 minutes each	MD
Byaruhanga J.,<br>2020<br><br>[30]	n.58<br>TC	Computer-based internet video counseling<br>Mobile phone app (CASC)<br>Mobile carbon monoxide checker	5 counseling at 2, 4, 8, 12 and 24 weeks<br>MD<br>MD	24 weeks<br>MD<br>MD
Cartujano-Barrera F.,<br>2022<br><br>[31]	n.18<br>TC	Telephone counseling	5 counseling	MD
Chhabra D.,<br>2023<br><br>[32]	n.1319<br>TC	Website resources (WebQuit.org)	MD	57 days
	n.1318<br>TC	Website resources (Smokefree.gov)	MD	MD
	n.1214<br>TC	Mobile phone App (iCanQuit)	MD	MD
	n.1201<br>TC	Mobile phone App (QuitGuide)	MD	MD
	n.586<br>TC	Telephone counseling	5 counseling	MD
	n.584<br>TC	Quitline	5 sessions	MD
	n.143<br>TC	Mobile phone App (SmartQuit)	MD	22 days
	n.182<br>TC	Mobile phone App (QuitGuide)	MD	22 days
	n.49<br>TC	Website-based digital avatar counseling and exercise (Flexiquit)	6 counseling 25 minutes each	MD
Cobos-Campos R.,<br>2020<br><br>[33]	n.633<br>TC	Mobile phone App (MobileQuit)	MD	6 months
	n.638<br>TC	Computer App (QuitOnline)	MD	6 months
	n.820<br>TC	Mobile phone App (Crush the Crave)	MD	MD
do Amaral L.M.,	n.814	Quitline	MD	MD

2020<br><br>[34]	TC			
	n.46<br>TC	Institutional video during hospitalization	MD	N/D
	n.805<br>TC	Telephone counseling	2 weeks after discharge, and at 1-3-7-14-30-42 days	42 days
Eghdami S.,<br>2023<br><br>[35]	n.208<br>TC	Mobile phone App (SmokeFree28) including advice, gamification	MD	MD
	n.217<br>TC	Mobile phone App with minimum components	MD	MD
	n.241<br>TC	Website resources (psychoeducational) (Smokefree.gov) by the National Cancer Institute	MD	MD
	n.262<br>TC	Tailored website resource<br>Mobile phone text-message<br>Email	1-5 text-message/day<br>MD<br>MD	MD<br>MD<br>MD
Fang Y.E.,<br>2023<br><br>[36]	n.80<br>TC	Telephone counseling	MD	MD
	n.311<br>TC	Website resources<br>Mobile phone text-message	MD<br>MD	MD<br>MD
	n.307<br>TC	Website resource	MD	MD
	n.633<br>TC	Mobile phone App (MobileQuit)	MD	MD
	n.638<br>TC	Computer App (QuitOnline)	MD	MD
	n.385<br>TC	Website resource	MD	MD
	n.395<br>TC	Mobile phone text-messages	MD	MD
	n.1214<br>TC	Mobile phone App (iCanQuit)	MD	MD
	n.1201<br>TC	Mobile phone App (QuitGuide)	MD	MD
Gainsbury S.,<br>2011<br><br>[37]	n.144<br>TC	Website resources<br>Mobile phone text-message<br>IVR<br>Email	MD<br>MD<br>Daily interaction<br>Daily	54 weeks
Graham A.L.,<br>2016	n.257<br>TC	Website-tailored (RealU Website) resources<br>Email	MD	20 weeks<br>30 weeks

[65]	n.260<br>TC	Standard government website resources (QuitNet.com)	MD	MD
	n.809<br>TC	Internet-based tailored resource	MD	MD
	n.422<br>TC	Mobile phone text-message	MD	MD
	n.755<br>TC	Internet-based tailored and not-tailored resource	MD	MD
	n.144<br>TC	Website resources<br>Mobile phone text-message<br>IVR<br>Email	MD<br>MD<br>Daily interaction<br>Daily	54 weeks
	n.190<br>TC	Website resource	MD	MD
	n.174<br>TC	Website resource	MD	MD
	n.202<br>TC	Website resoruce	MD	MD
	n.525<br>TC	Website resource (smokefree.gov with 16 external links)	MD	MD
	n.509<br>TC	Website resource (smokefree.gov with 16 external links)<br>Mobile text-message or quitline<br>Social media	MD	MD
	n.1029<br>TC	Interactive website resources<br>Tailored emails	MD<br>Up to 150	12 months<br>12 months
	n.1043<br>TC	Interactive website resources	MD	12 months
	n.562<br>TC	Internet-based resources which comprise six sections with related exercises to advance to the following section	MD	MD
	n.242<br>TC	Computer-based tailored counseling<br><br>Website resources<br>Computer-based message<br>Email	Up to 7 counseling (1 in the rehabilitation centre and 6 after discharge)<br>MD<br>MD<br>MD	6 months<br><br>6 months (concurrent)<br>6 months (concurrent)<br>MD
	n.877<br>TC	Tailored internet-based resource<br>Email with progress reports	MD<br>MD	MD<br>MD
	n.5404<br>TC	Website tailored resources (5 website: SmokeClinic, ORCAS, QuitNet, CAMH, and ProChange)	MD	MD

	n.1047<br>TC	Website resources minimally interactive	MD	MD
	n.81<br>TC	Website resources designed specifically for college students<br>Creation of a personal video message	MD<br>MD	MD<br>MD
	n.84<br>TC	Website resources	MD	MD
	n.552<br>TC	Website resources<br>Email	MD<br>MD	6 months<br>6 months
	n.139<br>TC	Internet-based resource ("Guía") by the National Cancer Institute<br>Internet-based lessons	MD<br>8 lessons available for 1 week	MD<br>1 week
	n.141<br>TC	Static website-based resource ("Guía" was adapted as a web-based brochure)	MD	MD
	n.142<br>TC	Internet-based resource ("Guía") by the National Cancer Institute<br>Internet-based lessons	MD<br>8 lessons available for 1 week	MD<br>1 week
	n.146<br>TC	Static website-based resource ("Guía" was adapted as a web-based brochure)	MD	MD
	n.651<br>TC	Website tailored and interactive resources (QuitNet.com)<br>Website-based group network	Patient choice<br>MD	6 months<br>MD
	n.679<br>TC	Website resources	MD	6 months
	n.720<br>TC	Static website resources	MD	MD
	n.670<br>TC	Video materials	MD	4 months
	n.708<br>TC	Computer based text-messages	MD	4 months
	n.2321<br>TC	Interactive tailored website resources	MD	MD
	n.2292<br>TC	Static one-page website resources	MD	MD
	n.1865<br>TC	Website resource	MD	MD
Hutton H.E., 2011<br><br>[66]	n.144<br>TC	Website resources<br>Mobile phone text-message<br>IVR<br>Email	MD<br>MD<br>Daily interaction<br>Daily	54 weeks
	n.3246<br>TC	Static website resource	MD	MD

	n.2158<br>TC	Interactive website resource	MD	MD
	n.139<br>TC	Internet-based resource ("Guía") by the National Cancer Institute<br>Internet-based lessons	MD<br>8 lessons available for 1 week	MD<br>1 week
	n.141<br>TC	Static website-based resource ("Guía" was adapted as a web-based brochure)	MD	MD
	n.142<br>TC	Internet-based resource ("Guía") by the National Cancer Institute<br>Internet-based lessons	MD<br>8 lessons available for 1 week	MD<br>1 week
	n.146<br>TC	Static website-based resource ("Guía" was adapted as a web-based brochure)	MD	MD
	n.251<br>TC	Internet-based resource ("Guía") by the National Cancer Institute<br>Email with links to the Guía sections to quit date	MD<br>MD	MD<br>MD
	n.247<br>TC	Internet-based resource ("Guía") by the National Cancer Institute	MD	MD
Iaccarino J.M., 2019<br><br>[38]	n.85<br>TC	Website resources (10 different website links)	Patient's choice	MD
	n.642<br>TC	Computer-tailored materials based on the patient's smoking behaviors and history	MD	MD
Kant R., 2021<br><br>[67]	n.197<br>TC	NRT<br>Website resources<br>Mobile phone text-message<br>IVR<br>Quitline<br>Email	MD<br>MD<br>3 text-message/day<br>Daily interaction<br>24h/day<br>Daily	MD<br>54 weeks<br>MD<br>MD<br>From the 15th day<br>First 6 weeks
	n.552<br>TC	Website resources<br>Email	MD<br>MD	6 months<br>6 months
	n.190<br>TC	Website resource	MD	MD
	n.257<br>TC	Website-tailored (RealU Website) resources<br>Email	MD	20 weeks<br>30 weeks
	n.85<br>TC	Website resources (10 different website links)	Patient's choice	MD
	n.476<br>TC	Interactive internet-based resources by the Research Centre	MD	MD
	n.272<br>TC	Internet-based resources	MD	MD
	n.144<br>TC	Website resources<br>Mobile phone text-message	MD<br>MD	54 weeks

		IVR<br>Email	Daily interaction<br>Daily	
Krishnan N.,<br>2021<br><br>[39]	n.674<br>TC	Mobile phone tailored text-message (Happy Quit)	3-5 text-messages/day for 12 weeks	6 months
	n.284<br>TC	Mobile phone tailored text-message (Happy Quit)	3-5 text-messages/week for 12 weeks	6 months
	n.411<br>TC	Mobile phone text-message not related to smoking	1 text-message/week	6 months
	n.4000<br>TC	Mobile phone not-tailored text-message (Test to Quit China)	91 text-messages for 6 weeks (3/day for the 1st and 2nd weeks, 2/day for 3-5 weeks, 1/day for the 6th weeks	6 weeks
	n.4000<br>TC	Mobile phone not-tailored text-message (Test to Quit China)	1 text-message/week	6 weeks
Li S.,<br>2024<br><br>[40]	n.81<br>TC	Website resources designed specifically for college students<br>Creation of a personal video message	MD<br>MD	MD<br>MD
	n.84<br>TC	Website resources	MD	MD
	n.591<br>TC	Mobile phone text-message (WhatsApp)	MD	MD
	n.452<br>TC	Telephone counseling	5 counseling	8 weeks
	n.452<br>TC	Interactive internet-based resources (including optional text-message and video)	MD	MD
	n.299<br>TC	Tailored app-based mobile phone text message (iQuit)	0-2 text-message/day (mean 1.2)	3 months
	n.81<br>TC	Mobile phone App (SmokeFree)<br>Mobile carbon monoxide checker	MD<br>MD	3 weeks<br>3 weeks
	n.190<br>TC	Website resource	MD	MD
	n.174<br>TC	Website resource	MD	MD
	n.202<br>TC	Website resource	MD	MD
	n.160<br>TC	Mobile phone text-message (SMsalud®)	2 text-messages/day for the first 5 weeks, 3 text-messages/week for the 6-26 weeks	MD
	n.78<br>TC	Website interactive and tailored for subjects with schizophrenia spectrum resources	1 session 30-90 minutes	MD
	n.84	Website static resources of the National Cancer Institute	1 session 30-90 minutes	MD

	TC			
	n.144<br>TC	Website resources<br>Mobile phone text-message<br>IVR<br>Email	MD<br>MD<br>Daily interaction<br>Daily	54 weeks
Lindson-Hawley N, 2016<br><br>[68]	n.164<br>TC	Telephone counseling<br>Tailored email	4 counseling (3 in the first 3 months)<br>5 emails (3 in the first 3 months)	6 months<br>6 months (concomitant)
	n.156<br>TC	Email	3 emails	6 months
Liu S.,<br>2017<br><br>[41]	n.299<br>TC	Tailored app-based mobile phone text message (iQuit)	0-2 text-message/day (mean 1.2)	3 months
	n.241<br>TC	Website resources (psychoeducational) (Smokefree.gov) by the National Cancer Institute	MD	MD
	n.262<br>TC	Tailored website resource<br>Mobile phone text-message<br>Email	1-5 text-message/day<br>MD<br>MD	MD<br>MD<br>MD
	n.144<br>TC	Website resources<br>Mobile phone text-message<br>IVR<br>Email	MD<br>MD<br>Daily interaction<br>Daily	54 weeks
Luo T.,<br>2021<br><br>[42]	n.299<br>TC	Tailored app-based mobile phone text message (iQuit)	0-2 text-message/day (mean 1.2)	3 months
	n.798<br>TC	Social media resources (Quitxt System)<br>Social media short text-message (Quitxt System)	MD<br>MD	MD<br>MD
	n.79<br>TC	Social media tailored resources (Facebook) with posts of US Public Health Services guidelines<br>Social media chat group (Facebook) moderated by the lead researcher	1 post/day for 90 days<br><br>1 session/week	MD<br><br>MD
Matkin W.,<br>2019<br><br>[69]	n.213<br>TC	Telephone counseling by a psychologist<br><br>Email	6 counseling<br><br>MD	At baseline, on quit date, 1, 3, 6 weeks, and 3 months<br>MD
McCrabb S.,<br>2019<br><br>[43]	n.977<br>TC	Computer-based tailored text-message	MD	MD
	n.1005<br>TC	Computer-based tailored text-message (one page shorter)	MD	MD
	n.1285<br>TC	Computer-based tailored resources	MD	MD

	n.1280<br>TC	Computer-based resources	MD	MD
	n.144<br>TC	Website resources<br>Mobile phone text-message<br>IVR<br>Email	MD<br>MD<br>Daily interaction<br>Daily	54 weeks
	n.2321<br>TC	Interactive tailored website resources	MD	>2 months
	n.2292<br>TC	Static one-page website resources	MD	MD
	n.190<br>TC	Website resource	MD	MD
	n.651<br>TC	Website tailored and interactive resources (QuitNet.com)<br>Website-based group network	Patient choice<br>MD	6 months<br>MD
	n.679<br>TC	Website resources	MD	6 months
	n.242<br>TC	Computer-based tailored counseling<br><br>Website resources<br>Computer-based message<br>Email	Up to 7 counseling (1 in the rehabilitation centre and 6 after discharge)<br>MD<br>MD<br>MD	6 months<br><br>6 months (concurrent)<br>6 months (concurrent)<br>MD
	n.164<br>TC	Website tailored resource (Decide2Quit)<br>Email	MD<br>MD	MD<br>MD
	n.299<br>TC	Website tailored resource (Decide2Quit)	MD	MD
	n.4097<br>TC	Static website resource<br>Email with links to smoking cessation guide	MD	MD
	n.4118<br>TC	Static website resource	MD	MD
	n.559<br>TC	Internet-based resources (text pages, booklets, testimonials)<br>Internet-based video<br>Internet-based chat group<br>Tailored text-messages	MD<br>MD<br>MD<br>MD	MD<br>MD<br>MD<br>MD
	n.561<br>TC	Internet-based resources (text pages, booklets, testimonials)<br>Internet-based video<br>Internet-based chat group	MD<br>MD<br>MD	MD<br>MD<br>MD
	n.562<br>TC	Internet-based resources which comprise six sections with related exercises to advance to the following section	MD	MD

	n.204<br>TC	Internet-based resource adapted by the Evolution Health Systems from the Stop Smoking Center with interim surveys	MD	MD
	n.199<br>TC	Internet-based resource adapted by the Evolution Health Systems from the Stop Smoking Center without interim surveys	MD	MD
	n.139<br>TC	Internet-based resource ("Guía") by the National Cancer Institute<br>Internet-based lessons	MD<br>8 lessons available for 1 week	MD<br>1 week
	n.141<br>TC	Static website-based resource ("Guía" was adapted as a web-based brochure)	MD	MD
	n.142<br>TC	Internet-based resource ("Guía") by the National Cancer Institute<br>Internet-based lessons	MD<br>8 lessons available for 1 week	MD<br>1 week
	n.146<br>TC	Static website-based resource ("Guía" was adapted as a web-based brochure)	MD	MD
	n.251<br>TC	Internet-based resource ("Guía") by the National Cancer Institute<br>Email with links to the Guía sections to quit date	MD<br>MD	MD<br>MD
	n.247<br>TC	Internet-based resource ("Guía") by the National Cancer Institute	MD	MD
	n.708<br>TC	Tailored video message	MD	MD
	n.132<br>TC	Tailored internet-based text-message	MD	MD
	n.81<br>TC	Website resources	MD	MD
Mersha A.G.,<br>2024<br><br>[44]	n.117<br>TC	Website (QuitNow Men) resources<br>Website-based chat group	MD	6 months
	n.31<br>TC	Social media resources (Facebook group)<br>Social media chat group (Facebook)	Post every 2-3 days	3 months
	n.79<br>TC	Social media tailored resources (Facebook) with posts of US Public Health Services guidelines<br>Social media chat group (Facebook) moderated by the lead researcher	1 post/day for 3 months<br><br>1 session/week	3 months<br><br>3 months
	n.81<br>TC	Mobile phone App (SmokeFree)<br>Mobile carbon monoxide checker	MD<br>MD	3 weeks<br>3 weeks
	n.79<br>TC	Social media tailored resources (Facebook) with posts of US Public Health Services guidelines<br>Social media chat group (Facebook) moderated by the lead researcher	1 post/day for 3 months<br><br>1 session/week	3 months<br><br>3 months
Naslund J.A.,<br>2017	n.79<br>TC	Social media tailored resources (Facebook) with posts of US Public Health Services guidelines	1 post/day for 3 months	3 months

[45]		Social media chat group (Facebook) moderated by the lead researcher	1 session/week	3 months
Nguyen A., 2023 [46]	n.221 TC	Telephone counseling<br>Mobile phone text-message	MD<br>MD	12 months<br>12 months (concurrent)
O'Logbon J., 2024 [47]	n.257 TC	Website-tailored (RealU Website) resources<br>Email	MD	20 weeks<br>30 weeks
	n.260 TC	Standard government website resources (QuitNet.com)	MD	MD
	n.81 TC	Website resources designed specifically for college students<br>Creation of a personal video message	MD<br>MD	MD<br>MD
	n.84 TC	Website resources	MD	MD
	n.82 TC	Nutrition website	MD	MD
Park E., 2023 [48]	n.820 TC	Mobile phone App (Crush the Crave)	MD	MD
	n.249 TC	Website resource (Smokefree.gov)	MD	MD
Piñeiro B., 2016 [49]	n.642 TC	Computer-tailored materials based on the patient's smoking behaviors and history	MD	MD
	n.85 TC	Website resources (10 different website links)	Patient's choice	MD
Ricker A.B., 2024 [50]	n.164 TC	Telephone counseling<br>Tailored email	4 counseling (3 in the first 3 months)<br>5 emails (3 in the first 3 months)	6 months<br>6 months (concomitant)
	n.156 TC	Email	3 emails	6 months
Saroj S.K., 2022 [51]	n.3631 TC	Telephone counseling<br>Mobile phone text-message	7 counseling at pre-quit day, quit day, 3, 7, 14, 21 and 30 days<br>7 text messages at 2, 3, 4, 5, 6, 9 and 12 months	30 days<br>12 months
	n.117 TC	Website (QuitNow Men) resources<br>Website-based chat group	MD	6 months
	n.1823 TC	Mobile phone tailored text-message	MD	MD
	n.1788 TC	e-mail message	MD	MD

	n.633<br>TC	Mobile phone App (MobileQuit)	MD	MD
	n.638<br>TC	Computer App (QuitOnline)	MD	MD
	n.342<br>TC	Mobile phone App with mandatory information about quitting options, daily motivational push notifications, quitting diary, and a quitting tracker	MD	MD
	n.342<br>TC	Mobile phone App with non-mandatory information about quitting options	MD	MD
Sawyer C.,<br>2023<br><br>[52]	n.2570<br>TC	Website resources (WebQuit Plus or Smokefree.gov)<br>Mobile phone text-message	MD<br>4 text-message/day	12 months<br>28 days
	n.78<br>TC	Website interactive and tailored for subjects with schizophrenia spectrum resources	1 session 30-90 minutes	
	n.84<br>TC	Website static resources of the National Cancer Institute	1 session 30-90 minutes	
Shahab L.,<br>2009<br><br>[53]	n.257<br>TC	Website-tailored (RealU Website) resources<br>Email	MD	20 weeks<br>30 weeks
	n.260<br>TC	Standard government website resources (QuitNet.com)	MD	MD
	n.197<br>TC	Website resources<br>NRT<br>Mobile phone text-message<br>IVR<br>Quitline<br>Email	MD<br>MD<br>3 text-message/day<br>Daily interaction<br>24h/day<br>Daily	54 weeks<br>MD<br>MD<br>MD<br>From the 15th day<br>First 6 weeks
	n.144<br>TC	Website resources<br>Mobile phone text-message<br>IVR<br>Email	MD<br>MD<br>Daily interaction<br>Daily	54 weeks
Spanakis P.,<br>2022<br><br>[54]	n.84<br>TC	Website static resources of the National Cancer Institute	1 session 30-90 minutes	MD
	n.78<br>TC	Website interactive and tailored for subjects with schizophrenia spectrum resources	1 session 30-90 minutes	MD
Staiger P.K.,<br>2020<br><br>[55]	n.820<br>TC	Mobile phone App (Crush the Crave)	MD	MD
Stead L.F.,<br>2017	n.51<br>TC	Telephone counseling	1 counseling of 10-15 minutes	N/D

[70]				
Tatnell P., 2022<br><br>[56]	n.49<br>TC	Website-based digital avatar counseling and exercise (Flexiquit)	6 counseling 25 minutes each	MD
Taylor G.M.J., 2017<br><br>[57]	n.257<br>TC	Website-tailored (RealU Website) resources<br>Email	MD	20 weeks<br>30 weeks
	n.260<br>TC	Standard government website resources (QuitNet.com)	MD	MD
	n.452<br>TC	Telephone counseling	5 counseling	8 weeks
	n.452<br>TC	Interactive internet-based resources (including optional text-message and video)	MD	MD
	n.190<br>TC	Website resource	MD	MD
	n.174<br>TC	Website resource	MD	MD
	n.202<br>TC	Website resoruce	MD	MD
	n.144<br>TC	Website resource<br>Mobile phone text-message<br>IVR<br>Email	MD<br>MD<br>Daily interaction<br>Daily	54 weeks
	n.422<br>TC	Mobile phone text-message	MD	MD
	n.755<br>TC	Internet-based tailored and not-tailored resource	MD	MD
	n.809<br>TC	Internet-based tailored resource	MD	MD
	n.651<br>TC	Website tailored and interactive resources (QuitNet.com)<br>Website-based group network	Patient choice<br>MD	6 months<br>MD
	n.679<br>TC	Website resources	MD	6 months
	n.562<br>TC	Internet-based resources which comprise six sections with related exercises to advance to the following section	MD	MD
	n.242<br>TC	Computer-based tailored counseling	Up to 7 counseling (1 in the rehabilitation centre and 6 after discharge)	6 months

		Website resources<br>Computer-based message<br>Email	MD<br>MD<br>MD	6 months (concurrent)<br>6 months (concurrent)<br>MD
	n.163<br>TC	Computer-based message tailored and interactive message	MD	MD
	n.670<br>TC	Video materials	MD	4 months
	n.708<br>TC	Computer based text-messages	MD	4 months
	n.224<br>TC	Tailored email	MD	MD
	n.234<br>TC	Not-tailored email	MD	MD
	n.81<br>TC	Website resources designed specifically for college students<br>Creation of a personal video message	MD<br>MD	MD<br>MD
	n.84<br>TC	Website resources	MD	MD
	n.82<br>TC	Nutrition website	MD	MD
	n.139<br>TC	Internet-based resource ("Guía") by the National Cancer Institute<br>Internet-based lessons	MD<br>8 lessons available for 1 week	MD<br>1 week
	n.141<br>TC	Static website-based resource ("Guía" was adapted as a web-based brochure)	MD	MD
	n.142<br>TC	Internet-based resource ("Guía") by the National Cancer Institute<br>Internet-based lessons	MD<br>8 lessons available for 1 week	MD<br>1 week
	n.146<br>TC	Static website-based resource ("Guía" was adapted as a web-based brochure)	MD	MD
	n.251<br>TC	Internet-based resource ("Guía") by the National Cancer Institute<br>Email with links to the Guía sections to quit date	MD<br>MD	MD<br>MD
	n.247<br>TC	Internet-based resource ("Guía") by the National Cancer Institute	MD	MD
	n.5404<br>TC	Website tailored resources (5 website: SmokeClinic, ORCAS, QuitNet, CAMH, and ProChange)	MD	MD
	n.1047<br>TC	Website resources minimally interactive	MD	MD
	n.2321<br>TC	Interactive tailored website resources	MD	>2 months
	n.2292	Static one-page website resources	MD	MD

	TC			
Villanti A.C.,<br>2020<br><br>[58]	n.201<br>TC	Email	MD	MD
	n.249<br>TC	Website resource (Smokefree.gov)	MD	MD
	n.31<br>TC	Computer-based resources adapted from the Smoke Mall software	4 sessions	MD
	n.34<br>TC	Computer-based resources not related to smoking (diet, hypertension risk, stress)	4 sessions	MD
	n.81<br>TC	Website resources designed specifically for college students<br>Creation of a personal video message	MD<br>MD	MD<br>MD
	n.84<br>TC	Website resources	MD	MD
	n.82<br>TC	Nutrition website	MD	MD
	n.30<br>TC	Interactive website resource	MD	MD
	n.28<br>TC	Static website resource from the National Cancer Institute	MD	MD
	n.257<br>TC	Website-tailored (RealU Website) resources<br>Email	MD	20 weeks<br>30 weeks
	n.260<br>TC	Standard government website resources (QuitNet.com)	MD	MD
Whittaker R.,<br>2016<br><br>[59]	n.241<br>TC	Website resources (psychoeducational) (Smokefree.gov) by the National Cancer Institute	MD	MD
	n.262<br>TC	Tailored website resource<br>Mobile phone text-message<br>Email	1-5 text-message/day<br>MD<br>MD	MD<br>MD<br>MD
	n.30<br>TC	Mobile phone text-message not smoking related	1 text-message/day	2 months
	n.422<br>TC	Mobile phone text-message	MD	MD
	n.755<br>TC	Internet-based tailored and not-tailored resource	MD	MD
	n.299<br>TC	Tailored app-based mobile phone text message (iQuit)	0-2 text-message/day (mean 1.2)	3 months
	n.809<br>TC	Internet-based tailored resource	MD	MD

Whittaker R., 2019<br><br>[60]	n.299<br>TC	Tailored app-based mobile phone text message (iQuit)	0-2 text-message/day (mean 1.2)	3 months
	n.633<br>TC	Mobile phone App (MobileQuit)	MD	6 months
	n.638<br>TC	Computer App (QuitOnline)	MD	6 months
	n.143<br>TC	Mobile phone App (SmartQuit)	MD	22 days
	n.182<br>TC	Mobile phone App (QuitGuide)	MD	22 days
	n.342<br>TC	Mobile phone App with mandatory information about quitting options, daily motivational push notifications, quitting diary, and a quitting tracker	MD	MD
	n.342<br>TC	Mobile phone App with non-mandatory information about quitting options	MD	MD
	n.674<br>TC	Mobile phone tailored text-message (Happy Quit)	3-5 text-messages/day for 12 weeks	6 months
	n.284<br>TC	Mobile phone tailored text-message (Happy Quit)	3-5 text-messages/week for 12 weeks	6 months
	n.411<br>TC	Mobile phone text-message not related to smoking	1 text-message/week	6 months
	n.820<br>TC	Mobile phone App (Crush the Crave)	MD	MD
	n.4000<br>TC	Mobile phone not-tailored text-message (Test to Quit China)	91 text-messages for 6 weeks (3/day for the 1st and 2nd weeks, 2/day for 3-5 weeks, 1/day for the 6th weeks	6 weeks
	n.4000<br>TC	Mobile phone not-tailored text-message (Test to Quit China)	1 text-message/week	6 weeks
	n.30<br>TC	Mobile phone text-message not smoking related	1 text-message/day	2 months
	n.208<br>TC	Mobile phone App (SmokeFree28) including advice, gamification	MD	MD
	n.217<br>TC	Mobile phone App with minimum components	MD	MD
	n.241<br>TC	Website resources (psychoeducational) (Smokefree.gov) by the National Cancer Institute	MD	MD
	n.262<br>TC	Tailored website resource<br>Mobile phone text-message	1-5 text-message/day<br>MD	MD<br>MD

		Email	MD	MD
	n.160<br>TC	Mobile phone text-message (SMsalud®)	2 text-messages/day for the first 5 weeks, 3 text-messages/week for the 6-26 weeks	MD
	n.422<br>TC	Mobile phone text-message	MD	MD
	n.755<br>TC	Internet-based tailored and not-tailored resource	MD	MD
	n.809<br>TC	Internet-based tailored resource	MD	MD
Williams P.J.,<br>2023<br><br>[61]	n.85<br>TC	Website resources (10 different website links)	Patient's choice	MD
	n.171<br>TC	Telephone counseling	7 counseling, 1/week	7 weeks
	n.642<br>TC	Computer-tailored materials based on the patient's smoking behaviors and history	MD	MD
Zhou X.,<br>2023<br><br>[62]	n.820<br>TC	Mobile phone App (Crush the Crave)	MD	MD
	n.243<br>TC	Mobile phone text-message	3-5 text-message/days	6 weeks
	n.230<br>TC	Mobile phone text-message	MD	3 months
