# Supplementary material for: Effectiveness and Adherence of Standalone Digital Tobacco Cessation Modalities: A Systematic Review of Systematic Reviews
Source: Healthcare (Basel). 2025 Aug 26;13(17):2125. doi: 10.3390/healthcare13172125 (PMC12428045; doi:10.3390/healthcare13172125)
Supplement: Supplementary file 1 [file healthcare-13-02125-s001.zip › 18.05.25 Supplementary File S3.pdf]

Supplementary File S3:
Subgroups of the Digital Tobacco Cessation Modalities

3.3 Mobile Text Messaging: Qualitative Synthesis

3.3.1. Mobile text messaging

The smoking cessation strategy based on mobile text messaging was reported by eleven studies [28,34,36,39,40,51,57,59,60,62,65].
It was conducted on 27028 smokers [28,34,36,39,40,51,57,59,60,62,65], 317 of them were hospitalized patients for not defined disease [34].
The smoking cessation programs had a duration of 6 weeks for 16230 smokers [39,59,62], 2 months for 60 [59,60], 3 months for 243 [62], and 6 months for 4443 [28,39,59].
The 6 months CARs verified by cotinine test at 6 months were recorded in 2738 smokers and amounted to 138 (5.04%) former smokers [39,59].
The 6 months CARs self-reported at 6 months were recorded in 2498 smokers and amounted to 237 (9.49%) former smokers [28,40,59,62].
The 6 months CARs self-reported at 7 months were recorded in 1688 smokers and amounted to 104 (6.16%) former smokers [57,59,60,65].
The 12 months CARs self-reported at 12 months were recorded in 320 smokers and amounted to 78 (24.38%) former smokers [40,59].
The 7 days PPA verified by carbon monoxide tests at 6 months was recorded in 1509 smokers and amounted to 96 (6.36%) former smokers [40].
The 7 days PPA self-reported at 6 months was recorded in 20226 smokers and amounted to 5135 (25.39%) former smokers [28,36,39,40,59,60].
The 30 days PPA self-reported at 6 months was recorded in 317 smokers and amounted to 58 (18.30%) former smokers [34].
The 7 days PPA self-reported at 7 months was recorded in 1688 smokers and amounted to 340 (20.14%) former smokers [57,59,60,65].
The adherence rate was reported for 11118 subjects and was 83.76% at 6 months (the smoking status of 9312 subjects was assessed at 6 months follow-up) [39,40,59,60], and 84.36% (1424) for 1688 subjects at 7 months [57,59,60,65].
The satisfaction rate was assessed at 6 months in 320 subjects; more than 80% of them declared that they were satisfied or totally satisfied [40,59].
No other data were registered concerning these digital tobacco cessation modalities.

3.4 Smartphone Apps: Qualitative Synthesis

3.4.1. Smartphone Apps

The smoking cessation strategy based on smartphone app was reported by 15 studies [32,33,35,36,40-42,48,51,55,59,60,62].
It was conducted on 18286 smokers [32,33,35,36,40-42,48,51,55,59,60,62]; the mean age was reported for 5150 smokers and was 36.44 years old [26,32,33,40-42,48,51,55,59,60,62,63], while the gender ratio of 1 male to 1.06 female (4877 male and 5193 female) was registered for 10070 subjects [26,32,33,40-42,48,51,55,59,60,62,63].
The smoking status before the intervention was recorded for 3098 subjects who smoked a mean of 17.42 cigarettes per day [26,32,40-42,60,61,63].
The smoking cessation programs had a duration of 3 weeks for 1300 smokers [32,33,59,63], 3 months for 1798 [40-42,59,60], and 6 months for 2532 [33,36,51,59].
The 3 months CARs self-reported at 6 months were recorded in 1798 smokers and amounted to 465 (25.86%) former smokers [40-42,59,60].
The 6 months CARs verified by carbon monoxide test at 6 months were recorded in 1798 smokers and amounted to 189 (9.45%) former smokers [40-42,59,60].
The 6 months CARs self-reported at 6 months were recorded in 10070 smokers and amounted to 890 (8.84%) former smokers [26,32,33,35,36,40-42,48,51,55,59,60,62,63].

The 6.5 months CARs self-reported at 6.5 months were recorded in 850 smokers and amounted to 104 (12.24%) former smokers [35,59].
The 7 days PPA verified by carbon monoxide test at 6 months was recorded in 1600 smokers and amounted to 144 (9.00%) former smokers [14,26,59,63].
The 7 days PPA self-reported at 6 months was recorded in 7452 smokers and amounted to 1308 (17.55%) former smokers [26,33,36,48,51,55,59,62].
The 30 days PPA self-reported at 6 months was recorded in 4920 smokers and amounted to 504 (10.24%) former smokers [26,33,48,55,59,62].
The 7 days PPA self-reported at 6.5 months was recorded in 850 smokers and amounted to 134 (15.76%) former smokers [35,59].
The 7 days PPA self-reported at 12 months was recorded in 4830 smokers and amounted to 1316 (27.24%) former smokers [32,36].
The 30 days PPA self-reported at 12 months was recorded in 4830 smokers and amounted to 1036 (22.45%) former smokers [32,36].
The adherence rate was reported for 13456 subjects and was 43.17% at 6 months (the smoking status of 5809 subjects was assessed at 6 months follow-up) [26,32,33,35,36,40-42,48,55,51,59,60,62,63].
The satisfaction rate was assessed at 6 months in 4920 subjects, 351 of them declared that they used the smartphone app frequently, 340 found it easy to use, 337 found it well laid out, and 331 had confidence in using it [26,33,48,55,59,62].
No other data were registered concerning these digital tobacco cessation modalities.

3.4.2. Smartphone App plus Mobile Carbon Monoxide Checker

The smoking cessation strategy based on a smartphone app plus a mobile carbon monoxide checker was reported by two studies [40,44].
It was conducted on 162 smokers with a mean age of 31.32 years and a gender ratio of 1.31 male to 1 female (92 male and 70 female), smoking a mean of 13.07 cigarettes per day [40,44].
The smoking cessation programs had a duration of 3 weeks [40,44].
The 6 months CARs verified by carbon monoxide test at 6 months amounted to 36 (22.22%) former smokers [40,44].
No other data were registered concerning these digital tobacco cessation modalities.

3.5 Internet-based Websites and Programmes: Qualitative Synthesis

3.5.1. Website resources

The smoking cessation strategy based on website resources was reported by twenty-three studies [28,32,35,36,38,40,41,43,47-49,52-54,57-61,64-67].
It was conducted on 74848 smokers [28,32,35,36,38,40,41,43,47-49,52-54,57-61,64-67], 58 of them were affected by an undetermined mental illness [58] and 486 by schizophrenia [40,52,54]. The mean age was reported for 27932 subjects at 38.96 years [28,38,40,43,47,49,52,57,58,61,64-67], while the gender ratio of 28930 subjects was 1 male to 1.45 females (11824 males and 17106 females) [28,38,40,43,47,49,52,57,58,61,64-67].
The smoking status before the intervention was recorded for 4278 subjects who smoked a mean of 11.72 cigarettes per day [40,43,47,48,52,57,58,64-67].
The smoking cessation programs had a duration of 57-day for 1319 smokers [32], over 2 months for 9284 [28,43,57,65], 6 months for 2037 [43,57,65], and 12 months for 1043 [65].
The 6 months CARs verified by cotinine tests at 6 months were recorded for 964 smokers and amounted to 48 (4.98%) former smokers [35,41,59,60].
The 6 months CARs self-reported at 6 months were recorded for 3534 smokers and amounted to 388 (10.98%) former smokers [43,57,58,65].
The 6 months CARs verified by cotinine tests at 7 months were recorded for 18452 smokers and amounted to 1828 (9.91%) former smokers [28,43,57,65].
The 6 months CARs self-reported at 7 months were recorded for 6256 smokers and amounted to 552 (8.82%) former smokers [57,59,60,65].
The 12 months CARs self-reported at 12 months were recorded for 3703 smokers and amounted to 703 (18.98%) former smokers [40,43,57,67,65].

The 18 months CARs self-reported at 18 months were recorded in 2037 smokers and amounted to 87 (4.27%) former smokers [43,57,65].
The 24 months CARs self-reported at 24 months were recorded for 1926 smokers and amounted to 234 (12.15%) former smokers [38,49,61].
The 7 days PPA verified by carbon monoxide tests at 6 months was recorded for 1784 smokers and amounted to 180 (10.09%) former smokers [35,40,41,43,47,48,52,54,57-60,65].
The 7 days PPA self-reported at 6 months was recorded for 1182 smokers and amounted to 113 (9.56%) former smokers [36,43,48,58].
The 30 days PPA verified by carbon monoxide tests at 6 months was recorded for 420 smokers and amounted to 75 (17.86%) former smokers [40,47,57,58,65].
The 30 days PPA self-reported at 6 months was recorded for 1307 smokers and amounted to 137 (10.48%) former smokers [40,43,57].
The PPA verified by cotinine tests at 6 months was recorded in 964 smokers and amounted to 108 (11.20%) former smokers, but the time of verification was not defined [35,42,59,60].
The PPA self-reported at 6 months was recorded in 18452 smokers and amounted to 2760 (14.96%) former smokers, but the time of verification was not defined [28,43,57,65].
The 7 days PPA self-reported at 7 months was recorded in 5581 smokers and amounted to 1527 (27.36%) former smokers [47,52,57-60,64,65,67].
The 30 days PPA verified by carbon monoxide test at 7 months was recorded in 1820 smokers and amounted to 308 (16.92%) former smokers [47,52,57,58,64,65,67].
The 30 days PPA self-reported at 7 months was recorded in 1820 smokers and amounted to 420 (23.08%) former smokers [47,52,57,58,64,65,67].
The PPA verified at 7 months was recorded in 65 smokers and amounted to 17 (26.15%) former smokers, but the time and methods of verification were not defined [58].
The 30 days PPA self-reported at 9 months was recorded in 307 smokers and amounted to 71 (23.13%) former smokers [36].
The 7 days PPA self-reported at 11.5 months was recorded in 272 smokers and amounted to 102 (37.50%) former smokers [67].
The 30 days PPA self-reported at 11.5 months was recorded in 1686 smokers and amounted to 183 (10.85%) former smokers [43,57,65].
The 7 days PPA verified by carbon monoxide tests at 12 months was recorded for 838 smokers and amounted to 44 (5.25%) former smokers [38,48,49,58,61,67].
The 7 days PPA self-reported at 12 months was recorded for 12297 smokers and amounted to 1835 (14.92%) former smokers [32,43,48,57,58,65].
The 30 days PPA self-reported at 12 months was recorded for 4674 smokers and amounted to 874 (18.70%) former smokers [32,43,57,65].
The PPA verified by cotinine tests at 12 months was recorded in 952 smokers and amounted to 24 (2.52%) former smokers, but the time of verification was not defined [66,67].
The PPA self-reported at 12 months was recorded in 952 smokers and amounted to 56 (5.88%) former smokers, but the time of verification was not defined [66,67].
The 30 days PPA self-reported at 13 months was recorded in 12904 smokers and amounted to 1436 (11.13%) former smokers [57,64].
The PPA verified at 13 months was recorded in 5404 smokers and amounted to 594 (10.99%) former smokers, but the time and methods of verification were not defined [66].
The 30 days PPA self-reported at 18 months was recorded in 2037 smokers and amounted to 339 (16.64%) former smokers [43,57,65].
The 7 days PPA self-reported at 24 months was recorded in 1926 smokers and amounted to 255 (13.24%) former smokers [38,49,61].
The adherence rate was reported for 22359 subjects and was 73.76% at 6 months (the smoking status of 16491 subjects was assessed at 6 months follow-up) [28,35,40,41,43,47,52,54,57-60,65] for 8076 subjects at 7 months was 88.46% (the smoking status of 7144 subjects was assessed at 7 months follow-up) [47,52,57-60,64,65,67], for 1686 subjects at 11.5 months was 48.40% (the smoking status of 816 subjects was assessed at 11.5 months follow-up) [43,57,65], for 5853 subjects at 12 months was 61.28% (the smoking status of 3586 subjects was assessed at 12 months follow-up) [40,43,57,65,66], for 2037 subjects at 18 months was 68.58% (the smoking status of 1397 subjects was assessed at 18 months follow-up) [43,57,65].

The satisfaction rate was assessed in 486 smokers with schizophrenia using the Perceived Usefulness and Ease of Use Scale, obtaining a mean score of 8.59 at 6 months [40,52,54].
No other data were registered concerning these digital tobacco cessation modalities.

3.5.2. Computer App

The smoking cessation strategy based on computer App was reported by four studies [33,36,51,59].
It was conducted on 2552 smokers who smoked more than five cigarettes per day for at least 6 months [33,36,51,59].
The smoking cessation programs had a duration of 6 months [33,36,51,59].
The 7 days PPA self-reported at 6 months amounted to 492 (19.28%) former smokers [33,36,51,59].
The adherence rate was reported to be 33.86% at 6 months (the smoking status of 864 subjects was assessed at 6 months follow-up) [33,36,51,59].
No other data were registered concerning these digital tobacco cessation modalities.

3.5.3. Website resources plus Website-based group network

The smoking cessation strategy based on website resources plus website-based group network was reported by five studies [43,44,51,57,65].
It was conducted on 2187 [43,44,51,57,65]; the mean age and the gender ratio were reported for 234 smokers, with a mean age of 39.82 years in 234 males to 0 females [44,51].
The smoking status before the intervention was recorded for 2187 subjects, 1953 smoked more than five cigarettes per day [43,57,65], and 234 smoked more than 15 cigarettes per day for at least 24 years, and 48% of them had a minimally FTND score, 27% moderately, and 25% were highly dependent [44,51].
The smoking status after the programs was recorded in total for 102 subjects, 54 reduced the number of cigarettes smoked per day, and 48 did not change their smoking behavior [44,51].
The smoking cessation programs had a duration of 6 months for 2187 smokers [43,44,51,57,65].
The 18 months CARs self-reported at 18 months were recorded for 1953 smokers and amounted to 72 (3.69%) former smokers [43,57,65].
The PPA self-reported at 6 months were recorded for 234 smokers and amounted to 32 (13.68%) former smokers, but time was not specified [44,51].
The 30 days PPA self-reported at 12 months were recorded for 1953 smokers and amounted to 357 (18.28%) former smokers [43,57,65].
The 30 days PPA self-reported at 18 months were recorded for 1953 smokers and amounted to 387 (19.82%) former smokers [43,57,65].
The adherence rate was reported for 1953 subjects and was 74.0% (the smoking status of 1445 subjects was assessed at 6 months follow-up); 72.2% at 12 months (the smoking status of 1410 subjects was assessed at 12 months follow-up); 69.0% at 18 months (the smoking status of 1348 subjects was assessed at 18 months) [43,57,65].
No other data were registered concerning these digital tobacco cessation modalities.

3.5.4. Internet-based resources plus Internet-based lessons

The smoking cessation strategy based on internet-based resources plus internet-based lessons was reported by four studies [43,66,57,65].
It was conducted on 1124 smokers [43,66,57,65].
The 7 days PPA self-reported at 12 months amounted to 164 (14.59%) former smokers [43,66,57,65].
The adherence rate was 49.11% at 12 months (the smoking status of 552 subjects was assessed at 12 months follow-up) [43,66,57,65].

No other data were registered concerning these digital tobacco cessation modalities.

3.5.5. Internet-based resources plus Internet-based video plus Internet-based chat group

The smoking cessation strategy on internet-based resources plus internet-based video plus internet-based chat group was reported by one study [43].
It was conducted on 561 smokers [43].
The 30 days PPA self-reported at 6 months amounted to 86 (15.51%) former smokers [43].
No other data were registered concerning these digital tobacco cessation modalities.

3.5.6. Internet-based resources plus Internet-based video plus Internet-based chat group plus Internet-based text-message

The smoking cessation strategy on internet-based resources plus internet-based video plus internet-based chat group plus internet-based text-message was reported by one study [43].
It was conducted on 559 smokers [43].
The 30 days PPA self-reported at 6 months amounted to 95 (16.99%) former smokers [43].
No other data were registered concerning these digital tobacco cessation modalities.

3.5.7. Social media resources plus Social media text-message

The smoking cessation strategy based on social media resources plus social media text-message was reported by five studies [42,44,45,48,58].
It was conducted on 1568 smokers [42,44,45,48,58]; the mean age and the gender ratio were reported for 1035 subjects and amounted to 27.35 years and 1.65 males to 1 female (644 males and 391 females) [42,44,45].
The smoking status before the intervention was recorded for 502 subjects who smoked a mean of 10.8 cigarettes per day [48,58], while the smoking status after the intervention was recorded for 28 subjects who reduced the cigarettes smoked per day by ≥50% at 12 months [42,44,45].
The smoking cessation programs had a duration of 3 months for 237 smokers [42,44,45], while for the other 1331, the duration was not specified.
The 7 days PPA verified by cotinine or carbon monoxide tests at 6 months was recorded in 770 smokers and amounted to 38 (4.94%) former smokers [42,44,45,48,58].
The 7 days PPA self-reported at 6 months was recorded in 739 smokers and amounted to 92 (12.45%) former smokers [42,44,45,48,58].
The 1 day PPA at 7 months after the selected quit date was recorded in 798 smokers and amounted to 171 (21.43%) former smokers, but the methods of verification were not specified 654 [44].
The 7 days PPA verified by cotinine test at 12 months was recorded in 739 smokers and amounted to 40 (5.41%) former smokers [42,44,45,48,58].
The 7 days PPA self-reported at 12 months was recorded in 739 smokers and amounted to 104 (14.07%) former smokers [42,44,45,48,58].
The adherence rate was reported for 237 subjects and was 82.28% (the smoking status of 195 subjects was assessed at 6 months follow-up) and 72.15% at 12 months (the smoking status of 171 subjects was assessed at 12 months follow-up) [42,44,45].
No other data were registered concerning these digital tobacco cessation modalities.

3.6 AI-based interventions: Qualitative Synthesis

3.6.1. Quitline

The smoking cessation strategy based on quitline was reported by two studies [32,34].

It was conducted on 1398 smokers [32,34], 814 of them were hospitalized patients for not defined multiple diseases [34].

The smoking status before and after the intervention was recorded for 584 subjects who smoked more than five cigarettes per day for at least one month; the mean number of cigarettes smoked per day at the end of the intervention was reduced to 1.7 cigarettes per day [32].

The 30 days PPA self-reported at 6 months was recorded for 814 smokers and amounted to 265 (32.55%) former smokers [34].

The 7 days PPA self-reported at 12 months was recorded for 584 smokers and amounted to 181 (30.99%) former smokers [32].

The 30 days PPA self-reported at 12 months was recorded for 584 smokers and amounted to 168 (28.77%) former smokers [32].

No other data were registered concerning these digital tobacco cessation modalities.

3.6.2. Email

The smoking cessation strategy based on email was reported by five studies [50,51,57,58,68].

It was conducted on 2759 smokers [50,51,57,58,68], 312 of them were outpatients pre-surgery or diagnostic procedures [50,68]; the age range was reported only for 201 smokers and was 18-24 years old [58]; the gender ratio was reported only for 201 and was 1 male to 1.34 female (86 male and 115 female) [58].

The smoking status before the intervention was recorded for 971 subjects, 312 of them smoked at least twenty cigarettes per day and 58 (18.59%) of them reduced for more than 50% the number of cigarettes smoked per day at 12 months (15.3 cigarettes per day verified by carbon monoxide test) [50,68], while the mean number of cigarettes per day for the other 659 subjects was 19.28 cigarettes per day [57,58].

The smoking cessation programs had a duration of 6 months for 312 smokers [50,68], while for the other 2447, the duration was not specified.

The 7 days PPA self-reported at 6 months was recorded for 2447 smokers and amounted to 236 (9.64%) formers smokers [51,57,58].

The PPA at 12 months was recorded for 312 smokers and amounted to 14 (4.49%) former smokers, but the time and methods of verification were not specified [50,68].

The adherence rate was reported for 458 subjects and was 42.58% at 6 months (the smoking status of 195 subjects was assessed at 6 months follow-up) [57].

No other data were registered concerning these digital tobacco cessation modalities.

3.6.3. Chatbot

The smoking cessation strategy based on chatbot was reported by one study [27].

It was conducted on 205 smokers with a mean age of 32.8 years old and a gender ratio of 1.63 male to 1 female (127 male and 78 female) [27].

The PPA at 6 months amounted to 27 (13.17%) former smokers, but time and methods of verification were not specified [27].

No other data were registered concerning these digital tobacco cessation modalities.

3.6.4. Computer-based Text-messages

The smoking cessation strategy based on computer-based text-messages was reported by three studies [43,57,65].

It was conducted on 3693 smokers [43,57,65].

The 6 months CARs self-reported at 6 months were recorded in 1982 smokers and amounted to 55 (2.77%) former smokers [43].

The 6 months CARs self-reported at 12 months were recorded in 163 smokers and amounted to 14 (8.59%) former smokers [57].

The 12 months CARs self-reported at 12 months were recorded in 1548 smokers and amounted to 139 (8.98%) former smokers [43,57,65].

The adherence rate was reported for 1579 subjects and was 59.72% at 12 months (the smoking status of 943 subjects was assessed at 12-month follow-up) [43,57,65].
No other data were registered concerning these digital tobacco cessation modalities.

3.7 Other Digital Tobacco Cessation Modalities

3.7.1. Video materials

The smoking cessation strategy based on video materials was reported by four studies [34,43,57,65].
It was conducted on 2094 smokers [34,43], 46 of them were hospitalized patients for acute myocardial infarction [34].
The 12 months CARs self-reported at 12 months were recorded for 2048 smokers and amounted to 132 (6.45%) former smokers [43,57,65].
The 7 days PPA self-reported at 6 months were recorded for 46 smokers and amounted to 20 (43.48%) former smokers [34].
The 7 days PPA self-reported at 12 months were recorded for 46 smokers and amounted to 16 (34.78%) former smokers [34].
The adherence rate was reported for 1340 subjects and was 54.03% at 12 months (the smoking status of 724 subjects was assessed at 12 months follow-up) [57,65].
No other data were registered concerning these digital tobacco cessation modalities.

3.7.2. Video counseling

The smoking cessation strategy based on computer-based internet video counseling was reported by one study [29].
It was conducted on 201 smokers [29].
The 3 months CARs at 6 months amounted to 14 (6.97%) former smokers, but the methods of verification were not specified [29].
No other data were registered concerning these digital tobacco cessation modalities.

3.7.3. Telephone counseling

The smoking cessation strategy based on telephone counseling was reported by nine studies [29,31,32,34,36,40,57,62,70].
It was conducted on 2844 smokers [29,31,32,34,36,40,57,62,70], 805 of them were hospitalized patients for not defined multiple diseases [34], and 80 were affected by tuberculosis [36]; the mean age was recorded for 1015 smokers and was 55.87 years old [57,62,68], while the age range of the other 18 smokers was between 40 and 59 years old [31].
The smoking status before and after the intervention was recorded for 586 subjects who smoked more than five cigarettes per day for at least one month; the mean number of cigarettes smoked per day at the end of the intervention was reduced by 5.67 cigarettes per day [32].
The smoking cessation programs had a duration of more than 42 days for 805 smokers [34], 7 weeks for 171 [62], and 8 weeks for 904 smokers [68,57].
The 3 months CARs at 6 months were recorded for 229 smokers and amounted to 10 (4.37%) former smokers, but methods of verification were not specified [29].
The 6 months CARs verified by cotinine test at 6 months were recorded for 51 smokers and amounted to 6 (11.76%) former smokers [70].
The 6 months CARs self-reported at 6 months were recorded for 904 smokers and amounted to 78 (8.63%) former smokers [57,68].
The 12 months CARs self-reported at 12 months were recorded for 904 smokers and amounted to 66 (7.30%) former smokers [57,68].
The 30 days PPA self-reported at 6 months was recorded for 1727 smokers and amounted to 436 (25.25%) former smokers [31,34,57,68].
The PPA at 6 months was recorded for 80 smokers and amounted to 54 (67.50%) former smokers, but time and methods of verification were not specified [36].
The 7 days PPA self-reported at 12 months was recorded for 586 smokers and amounted to 173 (29.52%) former smokers [32].
The 30 days PPA verified by carbon monoxide test at 12 months was recorded for 171 smokers and amounted to 22 (12.87%) former smokers [62].

The 30 days PPA self-reported at 12 months was recorded for 757 smokers and amounted to 168 (22.19%) former smokers [32,62].
The adherence rate was reported for 904 subjects and was 78.98% at 12 months (the smoking status of 714 subjects was assessed at 12 months follow-up) [57,68], and for 51 subjects at 6 months was 94% (the smoking status of 48 subjects was assessed at 6 months follow-up) [70].
No other data were registered concerning these digital tobacco cessation modalities.

3.8 Combined Digital Tobacco Cessation Modalities: Qualitative Synthesis

3.8.1. Mobile Text Messaging plus Internet-based Websites and Programmes

3.8.1.1. Mobile text messaging plus Website resources

The smoking cessation strategy based on website resources plus mobile text messaging was reported by three studies [36,43,52].
It was conducted on 3334 smokers [36,43,52], 783 were affected by schizoaffective disorders [52], and 221 by bipolar disorders [52]; the mean age and the gender ratio were reported for 3023 smokers and were 47.07 years old, 1 male to 3.31 female (701 male and 2322 female), respectively [36,43,52].
The smoking cessation programs had a duration of 12 months for 2570 smokers [52], while for the other 764, the duration was not specified.
The 12 months CARs self-reported at 12 months were recorded for 453 smokers and amounted to 24 (5.30%) former smokers [43].
The 30 days PPA self-reported at 6 months was recorded for 2570 smokers and amounted to 451 (17.55%) former smokers [52].
The 30 days PPA self-reported at 9 months was recorded for 311 smokers and amounted to 72 (23.15%) former smokers [36].
The 30 days PPA self-reported at 12 months was recorded for 2570 smokers and amounted to 569 (22.14%) former smokers [52].
The adherence rate was assessed in the 221 subjects affected by bipolar disorders as the mean number of logins to the website resources, and amounted to 13.5 logins [52].
No other data were registered concerning these digital tobacco cessation modalities.

3.8.2. Mobile Text Messaging plus Other Digital Tobacco Cessation Modalities

3.8.2.1. Mobile Text Messaging plus Telephone counseling

The smoking cessation strategy based on telephone counseling plus mobile text messaging was reported by two studies [46,51].
It was conducted on 3852 smokers [46,51].
The smoking cessation programs had a duration of 12 months for all the 3852 subjects [46,51].
The 6 months CARs self-reported at 6 months were recorded for 3631 smokers and amounted to 566 (15.59%) former smokers [51].
The 9 months CARs self-reported at 9 months were recorded for 3631 smokers and amounted to 523 (14.40%) former smokers [51].
The 12 months CARs self-reported at 12 months were recorded for 3631 smokers and amounted to 465 (12.81%) former smokers [51].
The 30 days PPA verified by carbon monoxide or cotinine tests at 12 months was recorded for 221 smokers and amounted to 13 (5.88%) former smokers [46].
The 30 days PPA self-reported at 12 months was recorded for 221 smokers and amounted to 90 (40.72%) former smokers [46].
The adherence rate was assessed as the number of telephone counseling sessions completed. It was reported for 221 subjects, 117 (52.94%) of them completed all eight telephone counseling, 141 (63.80%) completed at least four telephone counseling [46].
No other data were registered concerning these digital tobacco cessation modalities.

3.8.3. Mobile Text Messaging plus Internet-based Websites and Programmes plus AI-based interventions

3.8.3.1. Mobile text messaging plus Website resources plus plus Social media plus Quitline

The smoking cessation strategy based on website resources plus mobile text messaging or quitline plus social media was reported by one study [65].
It was conducted on 509 smokers [65].
The 7 days PPA self-reported at 7 months amounted to 150 (29.47%) former smokers [65].
No other data were registered concerning these digital tobacco cessation modalities.

3.8.3.2. Mobile text messaging plus Website resources plus Email

The smoking cessation strategy based on website resources plus mobile text messaging plus email was reported by four studies [35,41,59,60].
It was conducted on 1048 smokers who smoked more than five cigarettes per day [35,41,59,60].
The 6 months CARs verified by cotinine test at 6 months amounted to 116 (11.07%) [35,41,59,60].
The PPA verified by cotinine test at 6 months amounted to 164 (15.65%) former smokers, but the time of verification was not specified [35,41,59,60].
The adherence rate was 72.14% at 6 months (the smoking status of 756 subjects was assessed at 6 months follow-up) [35,41,59,60].
No other data were registered concerning these digital tobacco cessation modalities.

3.8.3.3. Mobile text messaging plus Website resources plus IVR plus Email

The smoking cessation strategy based on website resources plus mobile text messaging plus IVR plus email was reported by nine studies [37,40,41,43,53,57,65-67].
It was conducted on 1296 smokers with a mean age of 39.5 years old and a gender ratio of 1 male to 1 female (648 male and 648 female), smoking a mean of 16.6 cigarettes per day [37,40,41,43,53,57,65-67].
The smoking cessation programs had a duration of 54 weeks [37,40,41,43,53,57,65-67].
The 12 months CARs self-reported at 12 months amounted to 261 (20.14%) former smokers [37,40,41,43,53,57,65-67].
The 7 days PPA self-reported at 6 months amounted to 378 (29.17%) former smokers [37,40,41,43,53,57,65-67].
The 7 days PPA self-reported at 12 months amounted to 423 (32.64%) former smokers [37,40,41,43,53,57,65-67].
The adherence rate was 81.94% at 12 months (the smoking status of 1062 subjects was assessed at 12 months follow-up) [37,40,41,43,53,57,65-67].
No other data were registered concerning these digital tobacco cessation modalities.

3.8.4. Smartphone App plus Other Digital Tobacco Cessation Modalities

3.8.4.1. Smartphone app plus Video counseling plus Mobile carbon monoxide checker

The smoking cessation strategy based on a smartphone app plus computer-based video counseling plus mobile carbon monoxide checker was reported by one study [30].
It was conducted on 58 smokers [30].
The 4 months CARs verified by a carbon monoxide checker at 6 months amounted to 43 (74.14%) former smokers [30].
The adherence rate was 98.28% (the smoking status of 57 was assessed) at 2 weeks, 1, 2, 3, and 6 months [30].
No other data were registered concerning these digital tobacco cessation modalities.

3.8.5. AI-based interventions plus Other Digital Tobacco Cessation Modalities

3.8.5.1. Email plus Telephone counseling

The smoking cessation strategy based on telephone counseling plus email was reported by three studies [50,68,69].

It was conducted on 541 smokers [50,68,69], 328 of them were outpatients pre-surgery or diagnostic procedures [50,68]; the range was reported only for 213 smokers and was 18-65 years old [69].

The smoking cessation status before the intervention was recorded for 328 subjects who smoked at least twenty cigarettes per day and 82 (25.00%) of them reduced for more than 50% the number of cigarettes smoker per day at 12 months (15.8 cigarettes per day verified by carbon monoxide test) [50,68].

The smoking cessation programs had a duration of 6 months for 328 smokers [50,68], while for the other 213, the duration was not specified.

The 1 day PPA self-reported at 12 months was recorded for 213 smokers and amounted to 18 (8.45%) former smokers [69].

The PPA at 12 months was recorded for 328 smokers and amounted to 22 (6.71%) former smokers, but the time and methods of verification were not specified [50,68].

The adherence rate was assessed as number of telephone counseling completed. It was reported for 213 subjects, 8 (3.76%) of them completed all the telephone counseling, 46 (21.60%) completed at least one telephone counseling [69].

No other data were registered concerning these digital tobacco cessation modalities.

3.8.6. Internet-based Websites and Programmes plus AI-based interventions

3.8.6.1. Website-based counseling and Exercise with a Digital Avatar

The smoking cessation strategy based on website-based counseling and exercise with a digital avatar was reported by two studies [32,56].

It was conducted on 98 smokers who smoked more than five cigarettes per day for at least one month, and reduced the mean number of cigarettes smoked per day to six cigarettes [32,56].

The 7 days PPA self-reported at 6 months amounted to 50 (51.02%) former smokers [32,56].

No other data were registered concerning these digital tobacco cessation modalities.

3.8.6.2. Website resources plus Email

The smoking cessation strategy based on website resources plus email was reported by nine studies [43,47,53,57,58,64-67].

It was conducted on 10074 smokers [43,47,53,57,58,64-67]; the mean age and the gender ratio were reported for 3932 smokers and were 32.55 years old, 1 male to 1.95 female (1335 male and 2597 female), respectively [47,53,57,58,64,65,67].

The smoking cessation status before the intervention was recorded for 2903 subjects who smoked a mean of 10.27 cigarettes per day [47,53,57,58,64,65,67].

The smoking cessation programs had a duration of 6 months for 1104 smokers [65,67] and 12 months for 1029 smokers [65].

The 3 months CARs self-reported at 6 months were recorded for 877 smokers and amounted to 56 (6.39%) former smokers [65].

The 20 weeks CARs self-reported at 6 months were recorded for 1104 smokers and amounted to 46 (4.16%) former smokers [65,67].

The 7 days PPA self-reported at 6 months was recorded for 1268 smokers and amounted to 117 (9.23%) former smokers [34,65,67].

The 7 days PPA self-reported at 7 months was recorded for 1799 smokers and amounted to 1064 (59.14%) former smokers [47,53,57,58,64,65,67].

The 30 days PPA verified by carbon monoxide test at 7 months was recorded for 1799 smokers and amounted to 595 (33.07%) former smokers [47,53,57,58,64,65,67].

The 30 days PPA self-reported at 7 months was recorded for 1799 smokers and amounted to 728 (40.47%) former smokers [47,53,57,58,64,65,67].
The 7 days PPA self-reported at 12 months was recorded for 6130 smokers and amounted to 595 (9.71%) former smokers [43,57,65,66].
The adherence rate was reported for 1981 subjects and was 52.09% (the smoking status of 1032 subjects was assessed at 6 months follow-up); 1799 subjects and was 93.00% at 7 months (the smoking status of 1673 subjects was assessed at 7 months follow-up) [43,57,65,66]; 1029 subjects and was 40.72% at 12 months (the smoking status of 419 subjects was assessed at 12 months) [4].
No other data were registered concerning these digital tobacco cessation modalities.

3.8.7. Internet-based Websites and Programmes plus Other Digital Tobacco Cessation Modalities

3.8.7.1. Website resources plus Creation of a personal video message

The smoking cessation strategy based on website resources plus the creation of a personal video message was reported by five studies [40,47,58,57,65].
It was conducted on 405 smokers with a mean age of 20.42 years and a gender ratio of 1.19 male to 1 female (220 male and 185 female) [40,47,58,57,65].
The 7 days PPA verified by carbon monoxide test at 6 months amounted to 130 (32.10%) former smokers [40,47,58,57,65].
The 30 days PPA verified by carbon monoxide test at 6 months amounted to 90 (22.22%) former smokers [40,47,58,57,65].
The adherence rate was 90.6% at 6 months (the smoking status of 367 subjects was assessed at 6 months follow-up) [40,47,58,57,65].
No other data were registered concerning these digital tobacco cessation modalities.

3.8.8. Internet-based Websites and Programmes plus AI-based interventions plus Other Digital Tobacco Cessation Modalities

3.8.8.1. Website resources plus Computer-based message plus Email plus Computer-based counseling

The smoking cessation strategy on computer-based counseling plus website resources plus computer-based message plus email was reported by three studies [43,57,65].
It was conducted on 726 smokers, all of whom were inpatient rehabilitation centres [43,57,65].
The smoking cessation programs had a duration of 6 months [43,57,65].
The 7 days PPA self-reported at 6 months amounted to 171 (23.55%) former smokers [43,57,65].
The adherence rate was 88.43% at 6 months (the smoking status of 639 subjects was assessed at 6 months follow-up) [43,57,65].
No other data were registered concerning these digital tobacco cessation modalities.

Table S3 clusters the data extracted for each individual digital tobacco cessation modality (intervention), Table S4 for each combined digital tobacco cessation modality (comparison), and Table S5 summarizes the overall individual vs. combined digital tobacco cessation modality data, as well as the overall of the data extracted.

Table S3. Individual Digital Tobacco Cessation Modalities: data extracted and clustered for each individual digital tobacco cessation modality (mobile text messaging, smartphone apps, internet-based and websites and programmes, AI-based interventions, other digital tobacco modalities): population features (sample, mean age, gender ratio, comorbidities); smoking behaviors (mean of smoked cigarettes/day and FTND before and after the digital tobacco cessation modalities); intervention's duration; effectiveness (CARs and PPA, biochemically verified and self-reported, sorted in chronological order); adherence (sorted in chronological order); satisfaction.

	Mobile Text Messaging	Smartphone Apps	Internet-based Websites and Programmes	AI-based interventions	Other Digital Tobacco Modalities
**Population Features**					
Sample	27028	18448	83399	8055	5139
Mean age	—	36.28 y.o.<br>(recorded for 5312/18448)	38.55 y.o.<br>(recorded for 29201/83399)	32.8 y.o.<br>(recorded for 205/8055)	55.87 y.o.<br>(recorded for 1015/5139)
Gender ratio	—	1M/1.06F<br>4969M/5263F<br>(recorded for 10232/18448)	1M/1.38F<br>12702M/17497F<br>(recorded for 30199/83399)	1.10M/1F<br>213M/193F<br>(recorded for 406/8055)	—
Comorbidities	Hospitalized for N/D disease:<br>317 (1.17%)	—	Psychiatric disorder:<br>544 (0.65%)	Outpatients pre-surgery or diagnostic procedure: 312 (3.87%)<br>Hospitalized for N/D disease: 814 (10.11%)	Tuberculosis: 80 (1.56%)<br>Hospitalized for N/D disease: 805 (15.66%)<br>Hospitalized for IMA: 46 (0.90%)
**Smoking Behaviors**					
Smoked cigarettes/ day (mean)<br>Before	—	17.2<br>(recorded for 3260/18448)	11.62<br>(recorded for 4780/83399)	11.21<br>(recorded for 1301/8055)	—
FTND (mean)<br>Before	—	—	Low: 112; Moderate: 63; High: 59<br>(recorded for 234/83399)	—	—
**Intervention Features**					
Duration Lenght	6w: 16230 (60.05%)<br>2m: 60 (0.22%)<br>3m: 243 (0.90%)<br>6m: 4443 (16.44%)	3w: 1462 (7.92%)<br>3m: 1798 (9.75%)<br>6m: 2532 (13.73%)<br>N/D: 12656 (68.60%)	7w: 1319 (1.58%)<br>2m: 9284 (11.13%)<br>3m: 237 (0.28%)<br>6m: 6776 (8.13%)	6m: 312 (3.87%)<br>N/D: 7743 (96.13%)	6w: 805 (15.66%)<br>7w: 171 (3.33%)<br>8w: 904 (17.59%)<br>N/D: 3259 (63.42%)

	N/D: 6052 (22.39%)		12m: 1043 (1.25%)<br>N/D: 64740 (77.63%)		
**Effectiveness: CARs** Former Smokers/Smokers Assessed (Former Smoker %)					
For 3m at 6m Self-reported	—	465/1798 (25.86%)	—	—	—
For 3m at 6m N/D methods	—	—	—	—	24/430 (5.58%)
For 6m at 6m Biochemically verified	138/2738 (5.04%)	225/1960 (11.48%)	48/964 (4.98%)	—	6/51 (11.76%)
For 6m at 6m Self-reported	237/2738 (9.49%)	890/10070 (8.84%)	388/3534 (10.98%)	55/1982 (2.77%)	78/904 (8.63%)
For 6m at 7m Biochemically verified	—	—	1828/18452 (9.91%)	—	—
For 6m at 7m Self-reported	104/1688 (6.16%)	—	552/6256 (8.82%)	—	—
For 6.5m at 6.5m Self-reported	—	104/850 (12.24%)	—	—	—
For 6m at 12m Self-reported	—	—	—	14/163 (8.59%)	—
For 12m at 12m Self-reported	78/320 (24.38%)	—	703/3703 (18.98%)	139/1548 (8.98%)	198/2952 (6.71%)
For 18m at 18m Self-reported	—	—	159/3990 (3.98%)	—	—
For 24m at 24m Self-reported	—	—	234/1926 (12.15%)	—	—
**Effectiveness: PPA** Former Smokers/Smokers Assessed (Former Smoker %)					
7d PPA at 6m Biochemically verified	96/1509 (6.36%)	144/1600 (9.00%)	218/2554 (8.54%)	—	—
7d PPA at 6m Self-reported	5135/20226 (25.39%)	1308/7452 (17.55%)	697/4473 (15.58%)	236/2447 (9.64%)	20/46 (43.48%)
30d PPA at 6m Biochemically verified	—	—	75/420 (17.86%)	—	—
30d PPA at 6m Self-reported	58/317 (18.30%)	504/4920 (10.24%)	318/2427 (13.10%)	265/814 (32.55%)	436/1727 (25.25%)
N/D time PPA at 6m Biochemically verified	—	—	108/964 (11.20%)	—	—
N/D time PPA at 6m Self-reported	—	—	2792/18686 (14.94%)	—	—
N/D methods and time at 6m	—	—	—	27/205 (13.17%)	54/80 (67.50%)
7d PPA at 6.5m Self-reported	—	134/850 (15.76%)	—	—	—
7d PPA at 7m Self-reported	340/1688 (20.14%)	—	1527/5581 (27.36%)	—	—
30d PPA at 7m Biochemically verified	—	—	308/1820 (16.92%)	—	—
30d PPA at 7m Self-reported	—	—	420/1820 (23.08%)	—	—
N/D methods and time at 7m	—	—	17/65 (26.15%)	—	—
30d PPA at 9m Self-reported	—	—	71/307 (23.13%)	—	—
7d PPA at 11.5m Self-reported	—	—	102/272 (37.50%)	—	—
30d PPA at 11.5m Self-reported	—	—	183/1686 (10.85%)	—	—
7d PPA at 12m Biochemically verified	—	—	84/1577 (5.33%)	—	—
7d PPA at 12m Self-reported	—	1316/4830 (27.24%)	2103/14160 (14.85%)	181/584 (30.99%)	189/632 (29.91%)
30d PPA at 12m Biochemically verified	—	—	357/1953 (18.28%)	—	22/171 (12.87%)
30d PPA at 12m Self-reported	—	—	874/4674 (18.70%)	—	168/757 (22.19%)
N/D time PPA at 12m Biochemically verified	—	—	24/952 (2.52%)	—	—

N/D time PPA at 12m Self-reported	—	—	56/952 (5.88%)	—	—
N/D methods and time at 12m	—	—	14/312 (4.49%)	—	—
30d PPA at 13m Self-reported	—	—	1436/12904 (11.13%)	—	—
N/D methods and time at 13m	—	—	594/5404 (10.99%)	—	—
30d PPA at 18m Self-reported	—	—	726/3990 (18.20%)	—	—
7d PPA at 24m Biochemically verified	—	—	255/1926 (13.24%)	—	—
**Adherence**					
At 6m	9312/11118 (83.76%)	—	18995/27101 (70.09%)	195/458 (42.58%)	48/51 (94.12%)
At 7m	1424/1688 (84.36%)	—	7144/8076 (88.46%)	—	—
At 11.5m	—	—	816/1686 (48.40%)	—	—
At 12m	—	—	5719/9167 (62.39%)	943/1579 (59.72%)	1438/2244 (64.08%)
At 18m	—	—	2745/3990 (68.80%)	—	—
**Satisfaction**					
At 6m	256/320 (80.00%) satisfied or totally satisfied	—	8.59 mean of the Perceived Usefulness and Ease of Use Scale in 486 subjects with schizophrenia	—	—

Acronyms: years old (y.o); male (M); female (F); percentage (%); no data available (—); not defined (N/D); Acute Myocardial Infarction (IMA); Fargström Test for Nicotine Dependence (FTND); Continuous Abstinence Rates (CARs); Point Prevalence Abstinence (PPA); days (d); weeks (w); months (m). In the "Effectiveness" section, the lines marked in grey highlight the biochemically assessed effectiveness rates, those in white self-report, and in those in light blue the method was not specified.

Table S4. Combined Digital Tobacco Cessation Modalities: data extracted and clustered for each combined digital tobacco cessation modality (every combination between mobile text messaging and/or smartphone apps and/or internet-based and/or websites and programmes and/or AI-based interventions and/or other digital tobacco modalities): population features (sample, mean age, gender ratio, comorbidities); smoking behaviors (mean of smoked cigarettes/day and FTND before and after the digital tobacco cessation modalities); intervention's duration; effectiveness (CARs and PPA, biochemically verified and self-reported, sorted in chronological order); adherence (sorted in chronological order).

	Mobile Text Messaging + Internet-based	Mobile Text Messaging + Other	Mobile Text Messaging + Internet-based + AI-based	Smartphone App + Other	AI-based + Other	Internet-based + AI-based	Internet-based + Other	Internet-based + AI-based Other
**Population Features**								
Sample	3334	3852	2853	58	541	10172	405	726
Mean age	47.07 (recorded for 3023/3334)	—	39.5 (recorded for 1296/2853)	—	—	32.55 y.o. (recorded for 3932/10172)	20.42 y.o. (recorded for 405/405)	—
Gender ratio	1M/3.31F 701M/2322F	—	1M/1F 648M/648F	—	—	1M/1.95F 1335M/2597F	1.19M/1F 220M/185F	—

	(recorded for 3023/3334)		(recorded for 1296/2853)			(recorded for 3932/10172)	(recorded for 405/405)	
Comorbidities	Psychiatric disorders: 1004 (30.11%)	—	—	—	Outpatients pre-surgery or diagnostic procedure: 541 (100%)	—	—	Inpatient rehabilitation centres 726 (100%)
**Smoking Behaviors**								
Smoked cigarettes/ day (mean) Before	—	—	16.6 (recorded for 1296/2853)	—	—	10.27 (recorded for 2903/10172)	—	—
After	—	—	—	—	15.8 (recorded for 82/541)	6.00 (recorded for 98/10172)	—	—
**Intervention Features**								
Duration Lenght	12m: 2570 (77.08%) N/D: 764 (22.92%)	12m: 3852 (100%)	54w: 1296 (45.43%) N/D: 1557 (54.57%)	—	6m: 328 (60.63%) N/D: 213 (39.37%)	6m: 1104 (10.85%) 12m: 1029 (10.12%) N/D: 8039 (79.03%)	—	6m: 726 (100%)
**Effectiveness: CARs** Former Smokers/Smokers Assessed (Former Smoker %)								
For 3m at 6m Self-reported	—	—	—	—	—	56/877 (6.39%)	—	—
For 4m at 6m Biochemically verified	—	—	—	43/58 (74.14%)	—	—	—	—
For 4.6m at 6m Self-reported	—	—	—	—	—	46/1104 (4.16%)	—	—
For 6m at 6m Biochemically verified	—	—	116/1048 (11.07%)	—	—	—	—	—
For 6m at 6m Self-reported	—	566/3631 (15.59%)	—	—	—	—	—	—
For 9m at 9 Self-reported	—	523/3631 (14.40%)	—	—	—	—	—	—
For 12m at 12m Self-reported	24/453 (5.30%)	465/3631 (12.81%)	261/1296 (20.14%)	—	—	—	—	—
**Effectiveness: PPA** Former Smokers/Smokers Assessed (Former Smoker %)								
7d PPA at 6m Biochemically verified	—	—	—	—	—	—	130/405 (32.10%)	—
7d PPA at 6m Self-reported	—	—	378/1296 (29.17%)	—	—	167/1366 (12.23%)	—	171/726 (23.55%)
30d PPA at 6m Biochemically verified	—	—	—	—	—	—	90/405 (22.22%)	—

30d PPA at 6m Self-reported	451/2570 (17.55%)	—	—	—	—	—	—	—
N/D time PPA at 6m Biochemically verified	—	—	164/1048 (15.65%)	—	—	—	—	—
7d PPA at 7m Self-reported	—	—	150/509 (29.47%)	—	—	1064/1799 (59.14%)	—	—
30d PPA at 7m Biochemically verified	—	—	—	—	—	595/1799 (33.07%)	—	—
30d PPA at 7m Self-reported	—	—	—	—	—	728/1799 (40.47%)	—	—
30d PPA at 9m Self-reported	72/311 (23.15%)	—	—	—	—	—	—	—
1d PPA at 12m Self-reported	—	—	—	—	18/213 (8.45%)	—	—	—
7d PPA at 12m Self-reported	—	—	423/1296 (32.64%)	—	—	595/6130 (9.71%)	—	—
30d PPA at 12m Biochemically verified	—	13/221 (5.88%)	—	—	—	—	—	—
30d PPA at 12m Self-reported	569/2570 (22.14%)	90/221 (40.72%)	—	—	—	—	—	—
N/D methods and time at 12m	—	—	—	—	22/328 (6.71%)	—	—	—
**Adherence**								
At 2w	—	—	—	57/58 (98.28%)	—	—	—	—
At 1m	—	—	—	57/58 (98.28%)	—	—	—	—
At 2m	—	—	—	57/58 (98.28%)	—	—	—	—
At 3m	—	—	—	57/58 (98.28%)	—	—	—	—
At 6m	—	—	756/1048 (72.14%)	57/58 (98.28%)	—	1032/1981 (52.09%)	367/405 (90.62%)	639/726 (88.43%)
At 7m	—	—	—	—	—	1673/1799 (93.00%)	—	—
At 12m	—	—	1062/1296 (81.94%)	—	—	419/1029 (40.72%)	—	—

Acronyms: years old (y.o); male (M); female (F); plus (+); percentage (%); no data available (—); not defined (N/D); Acute Myocardial Infarction (IMA); Fargström Test for Nicotine Dependence (FTND); Continuous Abstinence Rates (CARs); Point Prevalence Abstinence (PPA); days (d); weeks (w); months (m). In the "Effectiveness" section, the lines marked in grey highlight the biochemically assessed effectiveness rates, those in white self-report, and in those in light blue the method was not specified.

Table S5. Individual vs. Combined Digital Tobacco Cessation Modalities and Overall: data clustered for all individual vs. combined digital tobacco cessation modalities and overall.

	Individual Overall	Combined Overall	Overall (Individual plus Combined)
**Population Features**			
Sample	142069	21941	164010
Mean age	38.70 y.o. (recorded for 35733/142069)	38.11 y.o. (recorded for 8656/21941)	38.58 y.o. (recorded for 44389/164010)
Gender ratio	1M/1.28F<br>17884M/22953F<br>(recorded for 40837/142069)	1M/1.98F<br>2904M/5752F<br>(recorded for 8656/21941)	1M/1.38F<br>20788M/28705F<br>(recorded for 49493/164010)
Comorbidities	Tuberculosis: 80 (0.06%)	Psychiatric disorders: 1004 (4.58%)	Tuberculosis: 80 (0.05%)

	Psychiatric disorder: 544 (0.38%)<br>Outpatients pre-surgery or diagnostic procedure: 312 (0.22%)<br>Hospitalized for N/D disease: 1936 (1.36%)<br>Hospitalized for IMA: 46 (0.03%)	Outpatients pre-surgery or diagnostic procedure: 541 (2.47%)<br>Inpatient rehabilitation centres 726 (3.31%)	Psychiatric disorder: 1548 (0.94%)<br>Outpatients pre-surgery or diagnostic procedure: 853 (0.52%)<br>Hospitalized for N/D disease: 1936 (1.18%)<br>Hospitalized for IMA: 46 (0.03%)<br>Inpatient rehabilitation centres 726 (0.44%)
**Smoking Behaviors**			
Smoked cigarettes/ day (mean): Before	13.51 (recorded for 9341/142069)	12.22 (recorded for 4199/21941)	13.11 (recorded for 13540/164010)
After	—	10.46 (recorded for 180/21941)	10.46 (recorded for 180/164010)
FTND (mean)#<br>Before	Low: 112; Moderate: 63; High: 59 (recorded for 234/142069)	—	Low: 112; Moderate: 63; High: 59 (recorded for 234/164010)
**Intervention Features**			
Duration Lenght	3w: 1462 (1.03%); 6w: 17035 (11.99%); 7w: 1490 (1.05%); 8w: 904 (0.64%); 2m: 9344 (6.58%); 3m: 2278 (1.60%); 6m: 14063 (9.90%); 12m: 1043 (0.73%); N/D: 94450 (66.48%)	6m: 2158 (9.84%); 12m: 7451 (33.96%); 54w: 1296 (5.91%); N/D: 10571 (48.18%)	3w: 1462 (0.89%); 6w: 17035(10.39%); 7w: 1490 (0.91%);<br>8w: 904 (0.55%); 2m: 9344 (5.70%); 3m: 2278 (1.39%);<br>6m: 16221 (9.89%); 12m: 8494 (5.18%); 54w: 1296 (0.79%); N/D: 105021 (64.03%)
**Effectiveness: CARs**			
For 3m at 6m Self-reported	465/1798 (25.86%)	56/877 (6.39%)	521/2675 (19.48%)
N/D methods for 3m at 6m	24/430 (5.58%)	—	24/430 (5.58%)
For 4m at 6m Biochemically verified	—	43/58 (74.14%)	43/58 (74.14%)
For 4.6m at 6m Self-reported	—	46/1104 (4.16%)	46/1104 (4.16%)
For 6m at 6m Biochemically verified	417/5713 (7.30%)	116/1048 (11.07%)	533/6761 (7.88%)
For 6m at 6m Self-reported	1648/19228 (8.57%)	566/3631 (15.59%)	2214/22859 (9.69%)
For 6m at 7m Biochemically verified	1828/18452 (9.91%)	—	1828/18452 (9.91%)
For 6m at 7m Self-reported	656/7944 (8.26%)	—	656/7944 (8.26%)
For 6.5m at 6.5m Self-reported	104/850 (12.24%)	—	104/850 (12.24%)
For 9m at 9m Self-reported	—	523/3631 (14.40%)	523/3631 (14.40%)
For 6m at 12m Self-reported	14/163 (8.59%)	—	14/163 (8.59%)
For 12m at 12m Self-reported	1118/8523 (13.12%)	750/5380 (13.94%)	1868/13903 (13.44%)
For 18m at 18m Self-reported	159/3990 (3.98%)	—	159/3990 (3.98%)
For 24m at 24m Self-reported	234/1926 (12.15%)	—	234/1926 (12.15%)
**Effectiveness: PPA**			

7d PPA at 6m Biochemically verified	458/5663 (8.09%)	130/405 (32.10%)	588/6068 (9.69%)
7d PPA at 6m Self-reported	7396/34644 (21.35%)	716/3388 (21.13%)	8112/38032 (21.33%)
30d PPA at 6m Biochemically verified	75/420 (17.86%)	90/405 (22.22%)	165/825 (20.00%)
30d PPA at 6m Self-reported	1581/10205 (15.49%)	451/2570 (17.55%)	2032/12775 (15.91%)
N/D time PPA at 6m Biochemically verified	108/964 (11.20%)	164/1048 (15.65%)	272/2012 (13.52%)
N/D time PPA at 6m Self-reported	2792/18686 (14.94%)	—	2792/18686 (14.94%)
N/D methods and time at 6m	81/285 (28.42%)	—	81/285 (28.42%)
7d PPA at 6.5m Self-reported	134/850 (15.76%)	—	134/850 (15.76%)
7d PPA at 7m Self-reported	1867/7269 (25.68%)	1214/2308 (52.73%)	3081/9577 (32.17%)
30d PPA at 7m Biochemically verified	308/1820 (16.92%)	595/1799 (33.07%)	903/3619 (24.95%)
30d PPA at 7m Self-reported	420/1820 (23.08%)	728/1799 (40.47%)	1148/3619 (31.72%)
N/D methods and time at 7m	17/65 (26.15%)	—	17/65 (26.15%)
30d PPA at 9m Self-reported	71/307 (23.13%)	72/311 (23.15%)	143/618 (23.14%)
7d PPA at 11.5m Self-reported	102/272 (37.50%)	—	102/272 (37.50%)
30d PPA at 11.5m Self-reported	183/1686 (10.85%)	—	183/1686 (10.85%)
1d PPA at 12m Self-reported	—	18/213 (8.45%)	18/213 (8.45%)
7d PPA at 12m Biochemically verified	84/1577 (5.33%)	—	84/1577 (5.33%)
7d PPA at 12m Self-reported	3789/20206 (18.75%)	1018/7426 (13.71%)	4807/27632 (17.40%)
30d PPA at 12m Biochemically verified	379/2124 (17.84%)	13/221 (5.88%)	392/2345 (16.72%)
30d PPA at 12m Self-reported	1042/5431 (19.19%)	659/2791 (23.61%)	1701/8222 (20.69%)
N/D time PPA at 12m Biochemically verified	24/952 (2.52%)	—	24/952 (2.52%)
N/D time PPA at 12m Self-reported	56/952 (5.88%)	—	56/952 (5.88%)
N/D methods and time at 12m	14/312 (4.49%)	22/328 (6.71%)	36/640 (5.63%)
30d PPA at 13m Self-reported	1436/12904 (11.13%)	—	1436/12904 (11.13%)
N/D methods and time at 13m	594/5404 (10.99%)	—	594/5404 (10.99%)
30d PPA at 18m Self-reported	726/3990 (18.20%)	—	726/3990 (18.20%)
7d PPA at 24m Biochemically verified	255/1926 (13.24%)	—	255/1926 (13.24%)
**Adherence**			
At 2w	—	57/58 (98.28%)	57/58 (98.28%)
At 1m	—	57/58 (98.28%)	57/58 (98.28%)

At 2m	—	57/58 (98.28%)	57/58 (98.28%)
At 3m	—	57/58 (98.28%)	57/58 (98.28%)
At 6m	28550/38728 (73.72%)	2851/4218 (67.59%)	31401/42946 (73.12%)
At 7m	8568/9764 (87.75%)	1673/1799 (93.00%)	10241/11563 (88.57%)
At 11.5m	816/1686 (48.40%)	—	816/1686 (48.40%)
At 12m	8100/12990 (62.36%)	1481/2325 (63.70%)	9581/15315 (62.56%)
At 18m	2745/3990 (68.80%)	—	2745/3990 (68.80%)
**Satisfaction**			
At 6m	256/320 (80.00%) satisfied or totally satisfied; 8.59 mean of the Perceived Usefulness and Ease of Use Scale in 486 subjects with schizophrenia	—	256/320 (80.00%) satisfied or totally satisfied; 8.59 mean of the Perceived Usefulness and Ease of Use Scale in 486 subjects with schizophrenia

Acronyms: years old (y.o); male (M); female (F); plus (+); percentage (%); no data available (—); not defined (N/D); Acute Myocardial Infarction (IMA); Fargström Test for Nicotine Dependence (FTND); Continuous Abstinence Rates (CARs); Point Prevalence Abstinence (PPA); days (d); weeks (w); months (m). In the "Effectiveness" section, the lines marked in grey highlight the biochemically assessed effectiveness rates, those in white self-report, and in those in light blue the method was not specified.
