# Supplementary material for: Effectiveness and Adherence of Standalone Digital Tobacco Cessation Modalities: A Systematic Review of Systematic Reviews
Source: Healthcare (Basel). 2025 Aug 26;13(17):2125. doi: 10.3390/healthcare13172125 (PMC12428045; doi:10.3390/healthcare13172125)
Supplement: Supplementary file 1 [file healthcare-13-02125-s001.zip › 18.05.25 Supplementary File S4.pdf]

Supplementary File S4: Quality Assessment

A total of 45 systematic reviews were judged using the AMSTAR-2 tool as follows: 13 (28.89% of the included systematic reviews) [27,29,30,43,45,47,54,57,59,60,68-70] were "high quality", 4 (8.89%) [32,34,36,56] were "moderate quality", 11 (24.44%) [26,28,33,38-40,44,58,63,65,67] were "low quality", 17 (37.78%) [31,35,37,41,42,46,48-53,55,61,62,64,66] were "critically low quality".

Table S6. The quality assessment based on the AMSTAR-2 tool of the alphabetically ordered included systematic reviews. Critical domains, as defined by AMSTAR-2, are indicated by blue shading.

Included Systematic Reviews	1	2	3	4	5	6	7	8	9	10	11	12	13	14	15	16	Quality Assessment
Barnett A., 2020 [63]	Y	N	Y	Y	Y	Y	Y	PY	Y	N	Y	Y	Y	Y	Y	Y	Low
Barroso-Hurtado M., 2021 [26]	Y	Y	Y	Y	Y	Y	PY	Y	Y	N	NMA	NMA	N	Y	NMA	Y	Low
Bendotti H., 2023 [27]	Y	Y	Y	Y	Y	Y	Y	Y	Y	N	Y	Y	Y	Y	Y	Y	High
Boland V.C., 2018 [28]	Y	N	Y	PY	Y	Y	PY	PY	Y	N	Y	Y	Y	Y	Y	Y	Low
Brown J., 2013 [64]	Y	N	Y	Y	N	N	N	Y	Y	N	NMA	NMA	Y	N	NMA	N	Critically Low
Byambasuren O., 2023 [29]	Y	Y	Y	Y	Y	Y	Y	PY	Y	N	Y	Y	Y	Y	Y	Y	High
Byaruhanga J., 2020 [30]	Y	Y	Y	Y	Y	Y	PY	Y	Y	Y	Y	Y	Y	N	Y	Y	High
Cartujano-Barrera F., 2022 [31]	N	Y	Y	Y	Y	Y	N	Y	N	N	NMA	NMA	N	N	NMA	Y	Critically Low
Chhabra D., 2023 [32]	Y	Y	Y	Y	Y	Y	PY	Y	Y	N	NMA	NMA	Y	N	NMA	Y	Moderate
Cobos-Campos R., 2020 [33]	Y	N	Y	Y	N	N	PY	PY	Y	N	Y	Y	Y	Y	Y	N	Low
do Amaral .M, 2020 [34]	Y	Y	Y	Y	N	Y	Y	Y	PY	N	NMA	NMA	Y	N	NMA	N	Moderate
Eghdami S., 2023 [35]	Y	N	Y	Y	Y	Y	N	N	Y	N	Y	Y	Y	Y	Y	Y	Critically low
Fang Y.E., 2023 [36]	Y	Y	Y	Y	Y	Y	Y	N	Y	Y	Y	N	Y	Y	Y	Y	Moderate

Gainsbury S., 2011 [37]	Y	N	Y	PY	N	N	N	N	PY	N	NMA	NMA	N	N	NMA	Y	Critically Low
Graham A.L., 2016 [65]	Y	N	Y	PY	Y	Y	Y	Y	Y	Y	Y	Y	Y	Y	Y	N	Low
Hutton H.E., 2011 [66]	Y	N	Y	N	Y	N	N	Y	Y	N	Y	Y	Y	Y	Y	Y	Critically Low
Iaccarino J.M., 2019 [38]	Y	N	Y	Y	N	Y	Y	PY	Y	N	NMA	NMA	Y	Y	NMA	Y	Low
Kant R., 2021 [67]	Y	N	Y	PY	Y	N	PY	N	Y	N	Y	Y	Y	Y	Y	Y	Low
Krishnan N., 2021 [39]	Y	N	Y	Y	Y	Y	PY	Y	Y	N	NMA	NMA	Y	N	NMA	Y	Low
Li S., 2024 [40]	Y	N	Y	Y	Y	Y	PY	PY	Y	N	Y	Y	Y	Y	Y	Y	Low
Lindson-Hawley N., 2016 [68]	Y	Y	Y	Y	Y	Y	Y	Y	Y	Y	Y	Y	Y	Y	Y	Y	High
Liu S., 2017 [41]	Y	N	Y	N	N	N	N	N	PY	N	Y	N	N	Y	N	Y	Critically Low
Luo T., 2021 [42]	Y	N	Y	PY	N	N	PY	PY	N	N	NMA	NMA	N	N	NMA	Y	Critically Low
Matkin W., 2019 [69]	Y	Y	Y	Y	Y	Y	Y	Y	Y	Y	Y	Y	Y	Y	Y	Y	High
McCrabb S., 2019 [43]	Y	Y	Y	Y	Y	Y	PY	Y	Y	N	Y	Y	Y	Y	Y	Y	High
Mersha A.G., 2024 [44]	Y	Y	Y	PY	Y	Y	N	Y	Y	N	NMA	NMA	Y	N	NMA	Y	Low
Naslund J.A., 2017 [45]	Y	Y	Y	Y	Y	Y	Y	Y	PY	N	NMA	NMA	Y	Y	NMA	Y	High
Nguyen A., 2023 [46]	Y	N	Y	Y	Y	Y	PY	Y	Y	N	NMA	NMA	N	Y	NMA	Y	Critically Low
O'Logbon J., 2024 [47]	Y	Y	Y	PY	Y	Y	PY	Y	Y	Y	Y	Y	Y	Y	Y	Y	High
Park E., 2023 [48]	N	N	N	N	Y	Y	PY	PY	PY	N	NMA	NMA	Y	Y	NMA	Y	Critically Low
Piñeiro B., 2016 [49]	N	N	Y	PY	Y	N	PY	Y	N	N	NMA	NMA	N	N	NMA	Y	Critically Low
Ricker A.B., 2024 [50]	Y	Y	Y	N	Y	N	Y	PY	Y	N	NMA	NMA	N	N	NMA	N	Critically Low

Saroj S.K., 2022 [51]	N	N	N	PY	N	Y	PY	Y	N	N	NMA	NMA	N	N	NMA	N	Critically Low
Sawyer C. 2023 [52]	Y	Y	Y	PY	Y	Y	PY	PY	N	N	NMA	NMA	N	Y	NMA	Y	Critically Low
Shahab L., 2009 [53]	Y	N	Y	PY	N	N	N	PY	N	N	Y	Y	Y	Y	N	Y	Critically Low
Spanakis P., 2022 [54]	Y	Y	Y	PY	Y	Y	PY	Y	Y	N	Y	Y	Y	Y	Y	Y	High
Staiger P.K., 2020 [55]	Y	N	Y	PY	Y	Y	N	PY	Y	N	NMA	NMA	Y	N	NMA	Y	Critically Low
Stead L.F., 2017 [70]	Y	Y	Y	Y	Y	Y	Y	Y	Y	Y	Y	Y	Y	Y	Y	Y	High
Tatnell P., 2022 [56]	Y	Y	Y	Y	Y	Y	PY	Y	Y	N	NMA	NMA	Y	N	NMA	Y	Moderate
Taylor G.M.J, 2017 [57]	Y	Y	Y	Y	Y	Y	Y	Y	Y	Y	Y	Y	Y	Y	Y	Y	High
Villanti A.C., 2020 [58]	Y	N	Y	Y	Y	Y	Y	Y	Y	Y	NMA	NMA	Y	Y	NMA	Y	Low
Whittaker R., 2016 [59]	Y	Y	Y	Y	Y	Y	Y	Y	Y	N	Y	Y	Y	Y	Y	Y	High
Whittaker R., 2019 [60]	Y	Y	Y	Y	Y	Y	Y	Y	Y	Y	Y	Y	Y	Y	Y	Y	High
Williams P.J., 2023 [61]	Y	Y	Y	Y	Y	Y	PY	PY	Y	N	Y	Y	N	Y	N	Y	Critically Low
Zhou X., 2023 [62]	N	PY	Y	N	N	N	N	PY	Y	N	Y	Y	N	Y	Y	Y	Critically Low

Acronyms: Yes (Y); No (N); Partially Yes (PY); No Meta-Analysis (No MA).
